# BRD7 Inhibited Immune Escape in Nasopharyngeal Carcinoma via Inhibiting PD-L1 Expression

**DOI:** 10.7150/ijbs.103703

**Published:** 2025-02-10

**Authors:** Yilin Guo, Jiaxue Lu, Xiaoxu Li, Shiqi Yan, Jieyu Zhou, Ziying Tian, Ying Liu, Nan Li, Qing Zhou, Xiayu Li, Lei Shi, Su Jiang, Mengna Li, Xiao Zhou, Donghai Huang, Zhaoyang Zeng, Songqing Fan, Wei Xiong, Ming Zhou, Guiyuan Li, Wenling Zhang

**Affiliations:** 1 Department of Medical Laboratory Science, the Third Xiangya Hospital, Central South University, Changsha, Hunan, China.; 2 Department of Blood Transfusion, Children's Hospital Affiliated to Zhengzhou University,Henan Children's Hospital, Zhengzhou Children's Hospital, Zhengzhou, Henan, China.; 3 Department of Medical Laboratory Science, Xiangya School of Medicine, Central South University, Changsha, Hunan, China.; 4 Department of Clinical Laboratory, WuHu Hospital, East China Normal University (The Second People's Hospital, WuHu), Wuhu, Anhui, China.; 5 Department of Clinical Laboratory, Zhengzhou Orthopaedics Hospital, Zhengzhou, China.; 6 Department of Clinical Laboratory, First Affiliated Hospital of Guizhou University of Traditional Chinese Medicine, Guiyang, Guizhou, China.; 7 Hunan Key Laboratory of Nonresolving Inflammation and Cancer, Disease Genome Research Center, The Third Xiangya Hospital, Central South University, Changsha, Hunan, China.; 8 Department of Pathology, the Second Xiangya Hospital, Central South University, Changsha, Hunan, China.; 9 NHC Key Laboratory of Carcinogenesis, Hunan Key Laboratory of Oncotarget Gene, Hunan Cancer Hospital/ the Affiliated Cancer Hospital of Xiangya School of Medicine, Central South University, Changsha, Hunan, China.; 10 The Key Laboratory of Carcinogenesis and Cancer Invasion of the Chinese Ministry of Education, Central South University, Changsha, Hunan, China.; 11 Cancer Research Institute and School of Basic Medicine Sciences, Central South University, Changsha, Hunan, China.; 12 Department of Otolaryngology, Xiangya Hospital, Central South University, Changsha, Hunan, China.

**Keywords:** BRD7, Nasopharyngeal carcinoma, PD-L1, Immune escape

## Abstract

Therapeutic strategies aimed at harnessing anti-tumor immunity are being intensively investigated as they show promising results in cancer treatment. The PD-1/ PD-L1 pathway is an essential target for restoring functional anti-tumor immune response. BRD7 is a candidate tumor suppressor gene and nuclear transcription factor of nasopharyngeal carcinoma (NPC) which was cloned in our laboratory. In this paper, we reported that the candidate tumor suppressor gene BRD7 was strongly associated with good prognosis of NPC patients and negatively regulated PD-L1 expression. In addition, we found that BRD7 down-regulated PD-L1 expression and enhanced the killing function of CD8^+^ T lymphocytes in NPC cells through binding to p85α via the 485-651 domain and inhibiting the activity of PI3K, thereby inhibiting the activity of PI3K/AKT/mTOR/STAT3 pathway. *In vivo* experiments, the results showed that BRD7 could not only inhibit the growth of tumors, but also play a better anti-tumor effect when combined with PD-L1 antibody. These results provided further evidence that BRD7 inhibited immune escape of NPC through down-regulating PD-L1 expression.

## Introduction

Nasopharyngeal carcinoma (NPC) is a malignant epithelial tumor originating from the nasopharyngeal mucosa in the head and neck region, characterized by distinct geographic distribution, racial susceptibility, and high invasiveness and metastasis 1, 2. The incidence is mainly concentrated in Southeast Asia and southern China. Currently, the treatment of NPC still follows the principle of "radiotherapy as the mainstay, chemotherapy as an adjunct," but some patients still face issues such as poor efficacy, post-treatment recurrence, and/or metastasis 3. According to the 2022 Clinical Practice Guidelines for NPC by the Chinese Society of Clinical Oncology (CSCO), immunotherapy has become one of the recommended treatment options for NPC, with programmed cell death-1 (PD-1)/ programmed cell death-Ligand 1 (PD-L1) antibody therapy being the most representative 4-6. In China, NPC is mainly undifferentiated non-keratinizing carcinoma, with a PD-L1 positivity rate of over 95%, providing an important theoretical basis for anti-PD-1/PD-L1 antibody therapy for NPC 7-9.

Bromodomain-containing 7 (BRD7) is a member of the bromodomain-containing protein family. Previous studies in our research group had found that BRD7 was downregulated in NPC biopsy tissues and cell lines, confirming its role as a tumor suppressor gene and nuclear transcription factor in NPC, participating in various cellular processes such as cancer initiation and progression, glucose metabolism, spermatogenesis, chromatin remodeling, cell cycle, and transcriptional regulation 10-16. However, the relationship between BRD7 and immunotherapy in NPC is not yet clear, and whether it can serve as a new target for immunotherapy in NPC requires further exploration.

The PD-1/PD-L1 checkpoint axis is the most studied immune checkpoint axis in NPC 17. Under physiological conditions, the binding of PD-L1 on the surface of normal cells with PD-1 on the surface of T cells can induce immune tolerance, protecting normal cells. However, in the case of malignant tumors, upregulation of PD-L1 expression occurs due to factors such as high expression of MYC, EGFR, activation of the PI3K/AKT/mTOR signaling pathway, and the Ras/Raf/MEK/MAPK-ERK signaling pathway, leading to the interaction between PD-1 and its ligands PD-L1/PD-L2, thereby blocking the activation of immune cells, impairing T cells function, and serving as one of the main mechanisms by which tumor cells evade anti-tumor immunity 18-20. The high rate of PD-L1 positivity in NPC, attributed to factors such as EBV virus infection, indirectly indicates that NPC cells may evade immunity through the PD-1/PD-L1 axis, protecting tumor cells from immune attacks. In the past two years, PD-1/PD-L1 antibodies and combination therapies with chemotherapy have shown promising anti-tumor activity in NPC, with some projects entering Phase III clinical trials. However, due to the complex interactions between NPC cells and the tumor microenvironment (TME) and the unclear mechanisms of immune escape, the application of immune checkpoint inhibitors is somewhat limited, affecting the treatment outcomes of patients. Therefore, elucidating the key mechanisms of immune escape in the NPC microenvironment is of great significance for improving the efficacy of clinical immunotherapy. Additionally, combining anti-PD-1/PD-L1 therapy with other methods to enhance the efficacy of immunotherapy has become a feasible strategy. Currently, the regulatory relationship and mechanisms between BRD7 and PD-L1 in NPC, as well as the relationship between BRD7 and anti-tumor immunity, remain unclear; further exploration is needed to determine whether BRD7 can serve as a new therapeutic target to enhance the efficacy of immunotherapy.

In conclusion, this study aims to investigate the specific mechanism by which BRD7 regulates PD-L1 expression in NPC; elucidate the regulatory relationship between BRD7 and immune escape, and provide new insights and strategies for clinical immunotherapy of NPC.

## Materials and Methods

### Data acquisition

Microarray data for NPC were downloaded from the Gene Expression Omnibus (GEO, http://www.ncbi.nlm.nih.gov/geo/) database (GSE102349 and GSE12452). The GSE102349 dataset includes a total of 113 NPC tissues sequenced on the GPL11154 platform 21. The GSE12452 database consists of 31 NPC samples and 10 nasopharyngeal epithelium (NPE) samples sequenced on the GPL570 platform 22.

### Immunoinfiltrating cell analysis

Evaluate the infiltration of different immune cells using ssGSEA and CIBERSORT. Implement ssGSEA analysis through the GSVA package in R (version 4.3.0) 23. Calculate the Pearson correlation between 28 immune infiltrating cells and BRD7, followed by visualization using the "ggplot2" and "heatmap" packages. Analyze and validate the immune cell infiltration profile of the dataset using the CIBERSORT analysis platform (https://cibersort.stanford.edu/) and the LM22 file. Represent the differential levels of 22 immune-infiltrating cells using box plots.

### Cell lines, reagents, plasmids, and cell transfection

This study used the cell lines 5-8F, CNE2, and 293T. 5-8F and CNE2 are human nasopharyngeal undifferentiated squamous cell carcinoma cell lines, cultured in RPMI-1640 (Bio-Channel, China) complete medium containing 10% fetal bovine serum (FBS, Bio-Channel, China) and 1% penicillin-streptomycin solution, 100× (Genview, China). 293T is a human embryonic kidney cell line, cultured in DMEM (high glucose) (Bio-Channel, China) complete medium containing 10% FBS and 1% penicillin-streptomycin solution, 100×. All three cell lines were cultured at 37°C in a 5% CO_2_ cell culture incubator. The 5-8F and 293T cell lines were gifted by the Cancer Research Institute of Central South University; the CNE2 cell line was gifted by the Hunan Cancer Hospital. The BRD7 overexpression plasmid, control vector, PD-L1 overexpression plasmid, and control vector were purchased from Youbio Biotechnology (Hunan, China); the BRD7 knockdown plasmid and control vector were purchased from Genechem Biotechnology (Shanghai, China). Cells were seeded into 6-well plates at 4×10^5^ cells/well. After reaching 70% confluency, recombinant plasmids were transfected using NEOFECT™ DNA Transfection Reagent (Neofect Biotech, China) according to the manufacturer's protocol.

Anti-PD-L1 antibody Atezolizumab (GlpBio, USA, 5 µg/mL), the PI3K inhibitor LY294002 (MCE, USA, 35 µM) and the p-STAT3 inhibitor HO-3867 (Selleck, China, 7 µM) were added to cells.

### Nuclear and Cytoplasmic Extraction

The Nuclear and Cytoplasmic Extraction Kit was purchased from CWBIO (Jiangsu, China). The cells were washed with sterile PBS, leaving 1 mL in the culture flask. After scraping the cells with a cell scraper, the suspension was transferred to a 1.5 mL EP tube. Cells were collected by centrifugation at 1500 rpm for 15 mins. Nc-Buffer A (containing 1% Protease Inhibitor Cocktail (Biosharp, China)) was added to the collected cells, and the EP tube was vortexed and incubated on ice. Nc-Buffer B was then added in the corresponding ratio (Nc-Buffer A: Nc-Buffer B=1000: 55), vortexed, and incubated on ice. After centrifugation, the supernatant containing cytoplasmic protein was stored at -20 °C. Nc-Buffer C (containing 1% Protease Inhibitor Cocktail) was added to the pellet, vortexed, and incubated on ice. The sample was centrifuged, and the collected supernatant contained the nuclear protein.

### Chromatin immunoprecipitation (ChIP) assays

CNE2 and 5-8F cells were cross-linked in 1% formaldehyde for 10 mins at 37°C. DNA from fixed chromatin cells was immunoprecipitated using a ChIP assay kit (Beyotime, China) and BRD7 antibodies (Proteintech, USA) or Rabbit IgG antibodies (Beyotime, China) according to the manufacturer's protocol. The purified DNA was used for the following quantitative PCR (q-PCR). The PD-L1 promoter sequence was found through the NCBI websit, and two promoter primers were designed to ensure the experimental effect. The primers used to confirm whether BRD7 binds to the PD-L1 promoter in PCR are described in Table S1.

### q-PCR

Total RNA was extracted using TRIzol reagent (CWBIO, China), and the total RNA concentration and purity were assessed using NanoDrop™ One/One^C^. Reverse transcription was performed to synthesize cDNA using the HiFiScript cDNA Synthesis kit (CWBIO, China). SYBR Green qPCR Master Mix (Servicebio, China) was used for q-PCR. The fold change was calculated using the relative quantification method (2^-ΔΔCt^). The mRNA primer sequences are provided in Table S1.

### Western blot (WB)

Cells or tissues were lysed with RIPA lysis buffer (Biosharp, China), and protein concentrations were measured using a BCA protein assay kit (Biosharp, China). Protein samples (30 mg/well) were loaded on 10% SDS‒PAGE gels and then transferred to 0.22 μM PVDF membranes (PALL, USA). After blocking with skim milk, the membranes were incubated overnight at 4 °C in primary antibody followed by 1 hour of secondary antibody at 37 °C. The membranes were detected in the Bio-Rad Gel imaging analysis system scanner after incubated with ECL reagents (Biosharp, China). The primary antibodies involved in the test are shown in Table S2.

### Co-immunoprecipitation (Co-IP)

Transfected 5-8F, CNE2, and 293T cells were lysed with cell lysis buffer (Beyotime, China) containing protease inhibitors for 20 mins. The supernatant was collected into EP tubes after centrifugation at 12,000 rpm for 15 mins. Take 100 μL of the supernatant as input. The remaining supernatant was mixed with 20 μL of protein A+G agarose (Beyotime, China) and 1 μg of mouse IgG (Beyotime, China) at 4 °C for 2 hours for impurity removal. After centrifugation at 1000 g for 5 mins, collect the supernatant into a new EP tube. Add 4 μg of DYKDDDDK-tagged mouse polyAb (Proteintech, USA) to the supernatant and incubate overnight at 4 °C. Then, add 20 μL of agarose beads to the supernatant and mix at 4 °C for 3 hours. PBS was added to the samples and boiled at 100 °C for 5 mins. Finally, perform WB analysis.

### Flag-IP after peptide treatment

The three peptides were designed and synthesized by QYAOBIO (ChinaPeptides) (Table S4). The peptide powders were solubilized in 50 μL of 1% DMSO and then diluted to the desired concentrations with sterile PBS. Experiments were conducted in the 5-8F and CNE2 cell lines. First, the cells were seeded in 10 cm cell culture dishes and allowed to grow until they reached approximately 70% confluence. The cells were then transfected with a plasmid overexpressing Flag-GFP-tagged BRD7. After 24 hours, the medium was changed, and the three peptides (5 μM) were added to the culture for an additional 24 hours. Subsequently, the cells were collected using a cell scraper, and a Co-IP experiment was performed using Flag antibodies.

### Immunofluorescence assay

Transfected with the BRD7 plasmids for 48 hours, 5-8F and CNE2 cells were washed three times with PBS and fixed with 4% paraformaldehyde (Dingguo, China) for 30-50 mins at room temperature. Permeabilization was done with Immunostaining Permeabilization Buffer with Saponin (Beyotime, China) for 10 mins, followed by three 3-minute washes with PBS. Nonspecific binding sites were blocked with 5% BSA (Beyotim, China) for 30-60 mins. The cells were then incubated with the primary antibody overnight at 4 °C, followed by incubation with the secondary fluorochrome-labeled antibody for 1 hour at 37 °C. Finally, DAPI (Beyotime, China) was used to stain the nuclei, and cellular fluorescence was observed with an immunofluorescence microscope or laser-scanning confocal microscope.

### Extraction and expansion of primary T cells

Peripheral blood from healthy adults was used to isolate T lymphocytes. Peripheral blood mononuclear cells were separated using Ficoll separation solution (Cytiva, USA) and then added to 1640 complete culture medium with 10% FBS. T-Cell TransAct (Miltenyi, Germany) and 20 ng/mL IL-2 (ABclonal, China) were included to specifically stimulate and maintain the proliferation of T lymphocytes.

### Flow cytometry and coculture of tumor cells with T cells

T lymphocytes and tumor cells were cocultured at a ratio of 10:1 for 24 h. T cells were stained with an Annexin V-FITC/PI Apoptosis Detection Kit (Biosharp, China), APC Anti-Human CD3 Antibody (Elabscience, China), PerCP Anti-Human CD8a Antibody (Elabscience, China), and FITC Anti-Human CD279/PD-1 Antibody (Elabscience, China) according to the instructions. The percentage of apoptotic and PD-1-positive CD8^+^ T cells were counted and analyzed. The effect of BRD7 on the killing ability of T lymphocytes to tumor cells was determined using clone-forming and CCK8 after coculture; IFN-γ was detected using the Human IFN-γ ELISA Kit, and the absorbance was measured at 450 nm using an enzyme marker.

### Preparation of dendritic cells and specific T cells

*In vitro*, isolated PBMCs were added to 10% FBS-1640 medium containing 50 ng/mL GM-CSF (ABclonal, China) and 20 ng/mL IL-4 (ABclonal, China) for five days, and cell differentiation was observed under a light microscope. Then, 25 ng/mL IFN-γ was added. After one day of treatment, the cells were cocultured with the lysate of CNE2 cells for one day to activate dendritic cells. The prepared dendritic cells and expanded T cells were cocultured in 1640 complete medium supplemented with IL-2 for five days at a ratio of 1:5 so that they could produce specific T lymphocytes for CNE2. (Dendritic cells and T cells were extracted from the peripheral blood of the same person).

### Mouse models

Mice (BALB/c nude mice, female, 4 weeks old, 14-16 g each) were obtained from the Experimental Animal Center of Central South University and housed in SPF conditions. 5×10^6^ CNE2 cells transfected with plasmids were injected subcutaneously into the mice. After tumor formation, the mice were divided into different groups. Specific T cells recognizing CNE2, anti-PD-L1 antibody (5 mg/kg), LY-294002 (25 mg/kg, GlpBio, USA), and HO-3867 (25 mg/kg) were injected into the mice. At the end of the experiment, mice were euthanized, and tumor tissue was dissected, photographed, weighed, and subjected to IHC, WB, q-PCR, and other tests. Tumor volume was calculated (volume = length × width^2^ × 0.5), and a growth curve was plotted. (All experimental operations have passed the experimental animal welfare ethics review of Central South University, and the audit number is CSU-2023-0206).

### Clinical tissue samples and clinical data

The 20 NPC and 10 NPE tissue samples used for immunohistochemistry (IHC) were obtained from Xiangya Hospital of Central South University. In our previous study, tissue microarrays of different stages of NPC were made at 896 tissue sites. All NPC samples were collected from patients in the Department of Otolaryngology, Xiangya Hospital, Central South University, from January 2002 to October 2004 24. We followed up all patients with NPC and finally collected complete follow-up information for 50 patients. We asked them about their first episode time, treatment status, recurrence, and death time in detail and registered disease-free survival (DFS) and overall survival (OS) status and time with an Excel table. All data were statistically analyzed using a log-rank test, and P values were obtained.

### IHC

The tissue slices were dewaxed, rehydrated, and subjected to antigen retrieval with Tris-EDTA Antigen Repair Solution (Biosharp, China) at 95°C. After blocking endogenous peroxidase (ZSGB-BIO, China), the sections were incubated with 5% bovine serum albumin and then incubated with the primary antibody overnight at 4°C. Following PBS washing, the reaction enhancement solution (ZSGB-BIO, China) was incubated, and biotin-labeled secondary antibodies (ZSGB-BIO, China) were applied for 1 hour at room temperature, followed by DAB kit (ZSGB-BIO, China) treatment for 30 s-1 min. Hematoxylin was used for rapid staining.

For IHC-positive cell evaluation, a semiquantitative scoring system was utilized to assess staining intensity and positive areas. Staining intensity was graded from 0 to 3, while the proportion of stained cells was scored as 0 (negative), 1 (<25% positive), 2 (25%-50% positive), 3 (50%-75% positive), and 4 (>75% positive). The final score, ranging from 0 to 12, was calculated by multiplying these two scores. All sections were independently scored by two pathologists.

### Statistical analysis

The experimental results were statistically analyzed using GraphPad Prism 8.0, and the data were presented as mean ± standard error (Mean ± SD). All results with *P* < 0.05 were considered statistically significant. * indicates *P* < 0.05; ** indicates *P* < 0.01; *** indicates *P* < 0.001; **** indicates *P* < 0.0001; ns indicates no significant difference.

## Results

### The expression of BRD7 and PD-L1 was negatively correlated in NPC

**Figure 1:**
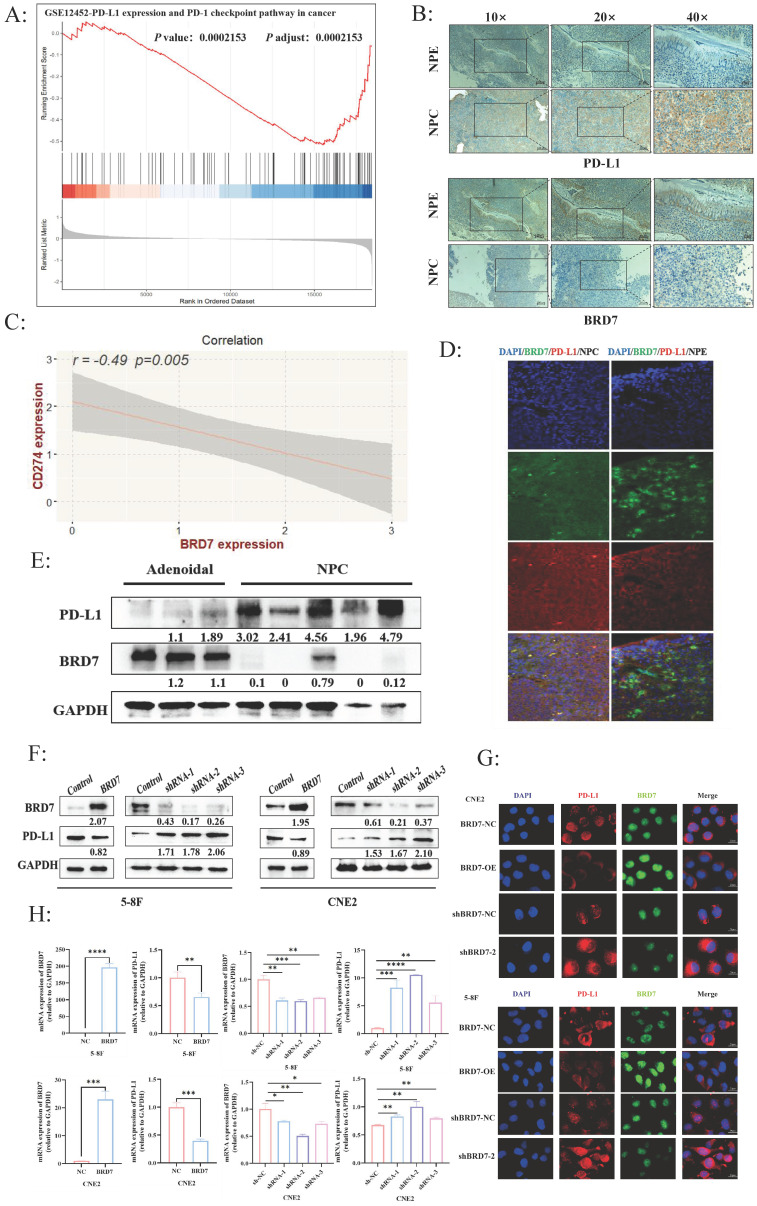
** The expression of BRD7 and PD-L1 was negatively correlated in NPC.** (A) GSEA showed that BRD7 was negatively correlated with PD-L1 expression (*P* =0.0002). (B) Representative images of IHC staining for BRD7 and PD-L1 expression in 10 NPE tissues and 20 NPC tissues. The scale bar is 20 μm. (C) Correlation analysis of IHC staining index between BRD7 and PD-L1 in 20 NPC tissues*. P* = 0.005, R = -0.49. (D) Representative images of multiplexed immunofluorescence of NPE and NPC tissues. Green fluorescence was BRD7, red was PD-L1, and nuclei were stained with DAPI (blue). NPE: nasopharyngeal epithelium; NPC: nasopharyngeal carcinoma. The scale bar is 20 μm. (E) The expression of BRD7 and PD-L1 in 3 adenoids and 5 samples of NPC were detected by western blot. (F) Western blot analysis of PD-L1 expression in NPC cell lines 5-8F and CNE2 stably transfected with BRD7 overexpression and knockdown or empty vector plasmids. (G) Representative immunofluorescence images for PD-L1 in 5-8F and CNE2 cells stably transfected with BRD7 overexpression and knockdown or empty vector plasmids. Red fluorescence was PD-L1, green fluorescence was BRD7, and nuclei were stained with DAPI (blue). The scale bar is 20 μm. (H) Relative PD-L1 mRNA levels measured by q-PCR in 5-8F and CNE2 cells stably transfected with BRD7 overexpression and knockdown or empty vector plasmids. *, *P* < 0.05; **, *P* < 0.01; ***, *P* < 0.001; ****, *P* < 0.0001; ns, not significant.

BRD7 is a candidate tumor suppressor gene cloned in our laboratory for NPC. In our preliminary studies, further validation through NPC tissue microarrays confirmed that BRD7 was underexpressed in NPC and closely associated with prognosis. Additionally, BRD7 was closely related to age (*P* = 0.011), gender (*P* = 0.007), and recurrence rate (*P* = 0.001) in NPC patients after radiotherapy (Table S3). A follow-up study was conducted on 50 NPC patients, revealing that BRD7-positive patients had better OS and DFS (Figure S1A). Based on these preliminary findings, we further explored the biological functions of BRD7 in NPC. To investigate the relationship between BRD7 and PD-L1 in NPC, we analyzed the GSE12452 dataset, which showed a negative correlation between BRD7 and PD-L1 expression (*P* < 0.001) (Figure 1A). Subsequently, immunohistochemical staining was performed on 10 NPE and 20 NPC tissues. The results indicated higher expression levels of BRD7 in NPE tissues compared to NPC tissues, while PD-L1 expression showed the opposite trend (Figure 1B). Correlation analysis of IHC scores revealed a negative correlation between BRD7 and PD-L1 expression (R = -0.49, *P* = 0.005) (Figure 1C). This conclusion was further supported by multiple fluorescence immunohistochemistry experiments (Figure 1D). Western blot experiments on clinical samples (3 nasopharyngeal adenoid and 5 NPC tissues) provided additional validation (Figure 1E). These results suggest a negative correlation between BRD7 and PD-L1 expression in NPC. To further investigate the relationship between BRD7 and PD-L1 in NPC cells, background expression of BRD7 was detected in several NPC cell lines, with the highest expression observed in CNE2 (Figure S1B). Subsequent experiments involved knocking down BRD7 expression in CNE2 cells and overexpressing it in 5-8F cells. Transient transfection of BRD7 overexpression or knockdown plasmids and their respective control plasmids into 5-8F and CNE2 cells resulted in significant changes in PD-L1 protein expression levels after 48 hours, as confirmed by Western blot and immunofluorescence experiments (Figure 1F-G, Figure S1C). Similarly, mRNA levels of BRD7 and PD-L1 were altered when BRD7 was overexpressed or knocked down in 5-8F and CNE2 cells (Figure 1H), indicating that BRD7 can downregulate PD-L1 expression in NPC cells.

### BRD7 downregulated PD-L1 expression and enhanced the cytotoxicity of T lymphocytes against NPC cells

**Figure 2:**
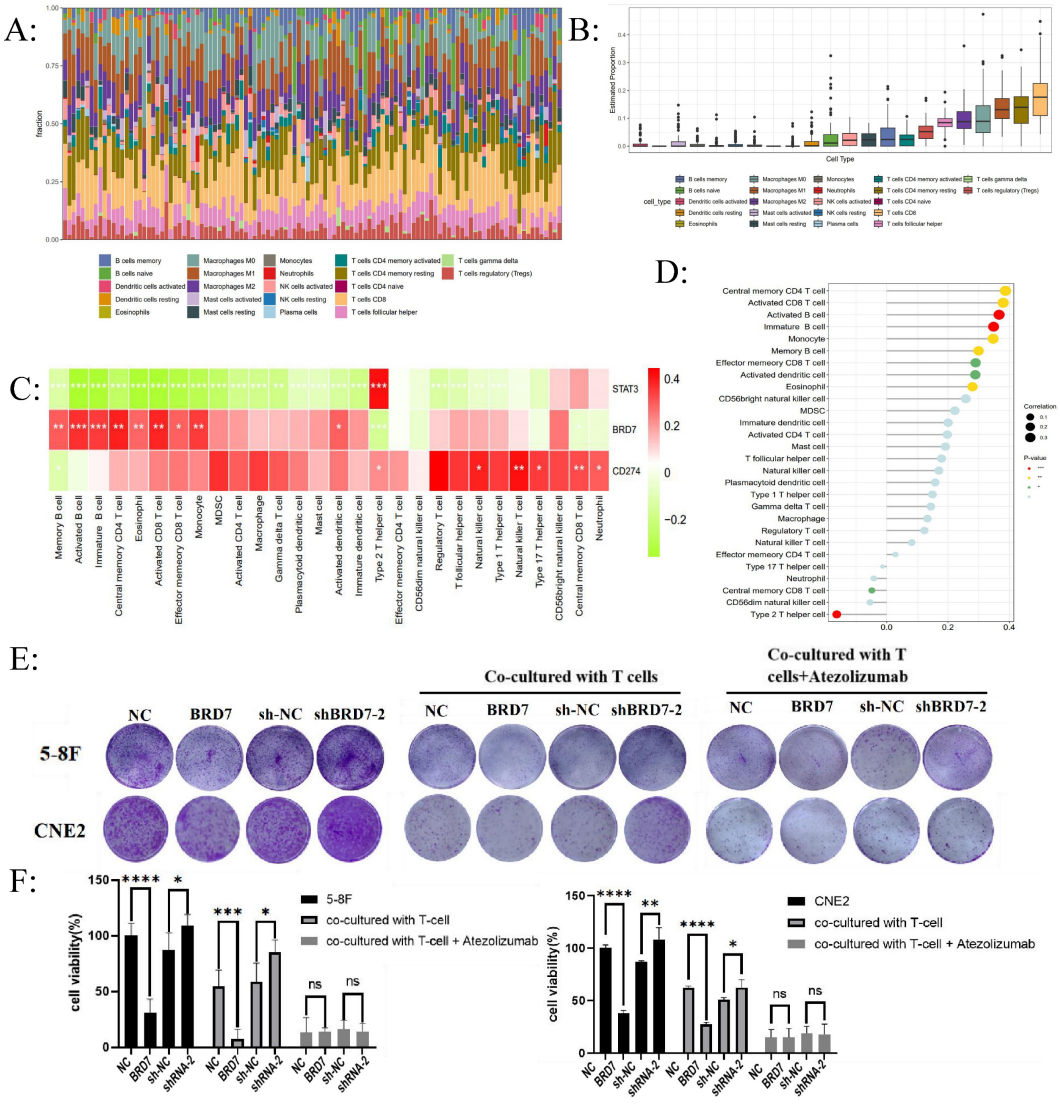

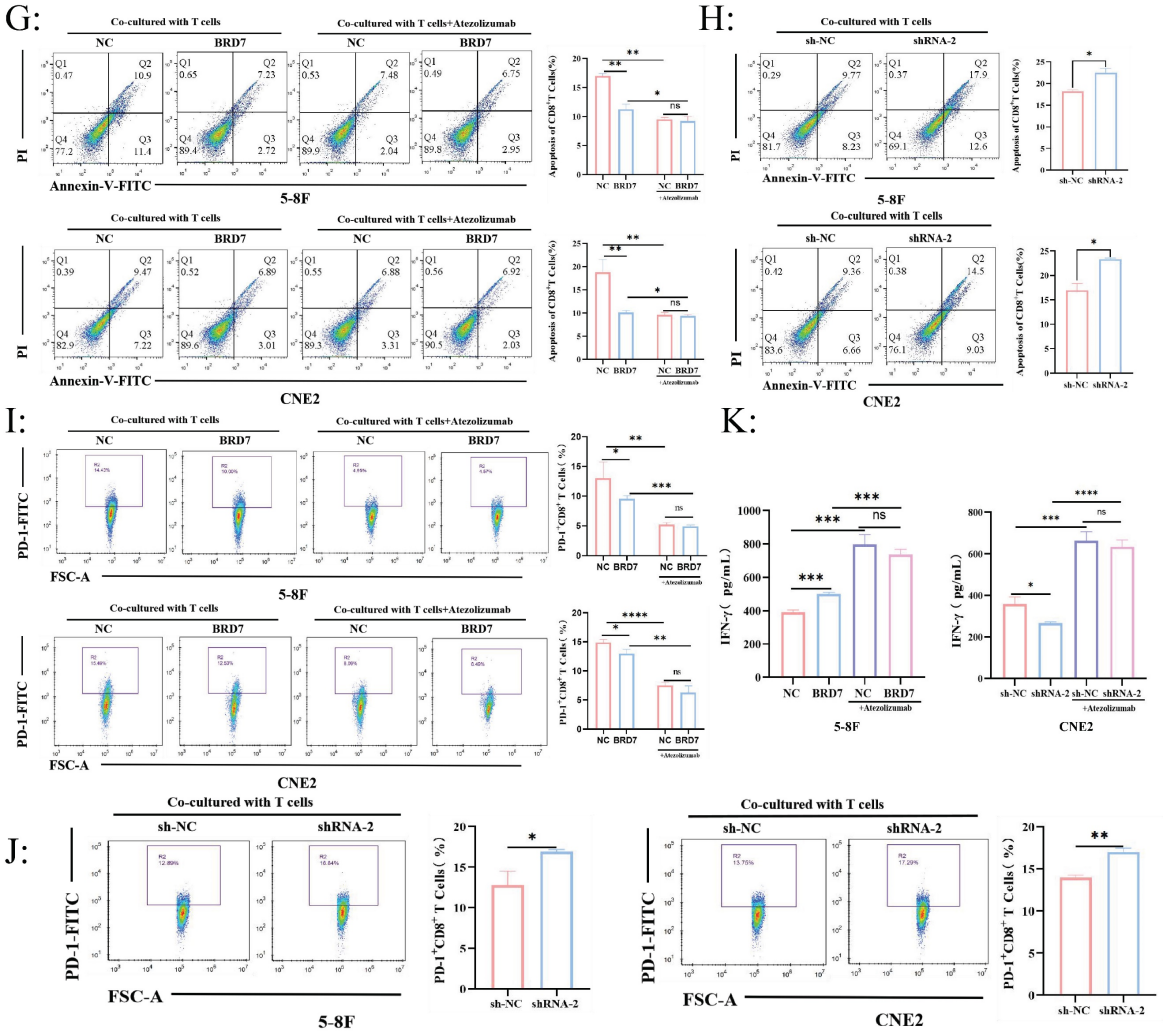
** BRD7 downregulated PD-L1 expression and enhanced the cytotoxicity of T lymphocytes against NPC cells.** (A) CIBERSORT determined the proportion of immune cell populations in NPC. (B) CD8^+^ T cell infiltration plays a vital role in NPC. (C) Correlation analysis between the expression of BRD7 and immune cells abundance. (D) Correlation analysis between BRD7 expression level and immune cell subtypes in GSE102349 of NPC. (E) Clonogenic assays of 5-8F and CNE2 cells stably transfected with BRD7 overexpression and knockdown or empty vector plasmids with or without T cells co-culture and PD-L1 antibody incubation. Atezolizumab: the PD-L1 antibody. (F) CCK-8 assay of 5-8F and CNE2 cells stably transfected with BRD7 overexpression and knockdown or empty vector plasmids with or without T cells co-culture and PD-L1 antibody incubation. Absorbance values were detected at 450 nm. *, *P* < 0.05; **, *P* < 0.01; ***, *P* < 0.001; ****, *P* < 0.0001; ns, not significant. (G) Flow cytometry detecting the apoptosis ratio of CD8^+^ T cells in T cells co-cultured with 5-8F or CNE2 cells stably transfected with BRD7 overexpression or empty vector plasmids with or without PD-L1 antibody incubation. (H) Flow cytometry detecting the apoptosis ratio of CD8^+^ T cells in T cells co-cultured with 5-8F or CNE2 cells stably transfected with BRD7 knockdown or empty vector plasmids. (I) Flow cytometry detecting the ratio of PD-1^+^ CD8^+^ T cells in T cells co-cultured with 5-8F or CNE2 cells stably transfected with BRD7 overexpression or empty vector plasmids with or without PD-L1 antibody incubation. (J) Flow cytometry detecting the ratio of PD-1^+^ CD8^+^ T cells in T cells co-cultured with 5-8F or CNE2 cells stably transfected with BRD7 knockdown or empty vector plasmids. (K) ELISA detecting the IFN-γ content in the culture medium of T cells co-cultured with 5-8F cells stably transfected with BRD7 overexpression or empty vector plasmids and CNE2 cells stably transfected with BRD7 knockdown or empty vector plasmids with or without PD-L1 antibody incubation for 24 hours. *, *P* < 0.05; **, *P* < 0.01; ***, *P* < 0.001; ****, *P* < 0.0001; ns, not significant.

Due to the close association between PD-L1 expression and the body's anti-tumor immune response, high expression of PD-L1 can weaken the killing ability of T cells on tumor cells through the PD-1/PD-L1 axis, leading to the phenomenon of "immune escape". We aimed to further investigate whether BRD7 may impact NPC immune escape by affecting PD-L1 expression. We evaluated the infiltration levels of different immune cells in NPC and the relationship between BRD7 and different immune cells using ssGSEA and CIBERSORT. The results indicated that CD8^+^ T lymphocyte infiltration predominates in NPC (Figure 2A-B).

Furthermore, among the 28 types of infiltrating immune cells in NPC, BRD7 was closely associated with the abundance of activated CD8^+^ T lymphocyte infiltration (Figure 2C-D). T lymphocytes and NPC cells were co-cultured at a ratio of 10:1 *in vitro*. The effects of BRD7 on tumor cell viability were assessed using crystal violet staining and CCK-8 experiments. The results indicated that BRD7 could inhibit the proliferation and viability of NPC cells and promote the killing ability of T cells (Figure 2E-F). Conversely, interfering with BRD7 promotes the proliferation of NPC cells, partially inhibiting the killing effect of T lymphocytes (Figure 2E-F). The addition of the PD-L1 antibody Atezolizumab in the co-culture system can block the PD-1/PD-L1 axis, further enhancing the killing effect of T lymphocytes on 5-8F and CNE2 cells.

To investigate the impact of BRD7 overexpression or interference in NPC cells on T lymphocyte function, we used flow cytometry to detect the apoptosis rate and PD-1 positivity of CD8^+^ T lymphocytes in the co-culture system supernatant. The results showed that when BRD7 was overexpressed, the apoptosis rate of CD8^+^ T lymphocytes and the proportion of PD-1^+^ CD8^+^ T cells decreased; upon adding the PD-L1 antibody, the apoptosis rate of CD8^+^ T lymphocytes and the proportion of PD-1^+^ CD8^+^ T cells in all groups decreased (Figure 2G-J). Conversely, when the expression of BRD7 was reduced, the apoptosis rate of CD8^+^ T lymphocytes and the proportion of PD-1^+^ CD8^+^ T cells increased (Figure 2G-J). IFN-γ, as a cytokine secreted by immunologically active cells, can indirectly reflect the activity of cytotoxic T lymphocytes. The results showed that the concentration of IFN-γ increased in the group with BRD7 overexpression, indicating a stronger killing function of T lymphocytes in the co-culture system (Figure 2K). When interfering with BRD7 expression, the concentration of IFN-γ in the supernatant decreased (Figure 2K). Similarly, upon adding the PD-L1 antibody Atezolizumab, the concentration of IFN-γ increased in all groups. These results suggested that BRD7 could inhibit the exhaustion of CD8^+^ T cells, enhance their killing function against tumor cells, and the addition of the PD-L1 antibody further inhibit this phenomenon, and could reverse the exhaustion of CD8^+^ T cells caused by downregulation of BRD7 expression.

We validated the effectiveness of the PD-L1 overexpression plasmid in two cell lines (Figure S2A-B). PD-L1 overexpression could reverse the downregulation of PD-L1 expression caused by BRD7 overexpression, and further promote the upregulation of PD-L1 expression caused by BRD7 downregulation (Figure S2C-D). Cell models with simultaneous overexpression of BRD7 and PD-L1 or simultaneous interference of BRD7 overexpression with PD-L1 were constructed in the 5-8F and CNE2 cell lines. The results indicated that PD-L1 overexpression could weaken the inhibitory effect of BRD7 on the growth of NPC cells and attenuate the inhibitory effect of BRD7 on CD8^+^ T cell exhaustion (Figure S2E-G). Combining the previous results, we draw a preliminary conclusion that in NPC, BRD7 could downregulate PD-L1 expression, weaken the PD-1/PD-L1 axis, thereby inhibiting the exhaustion of CD8^+^ T lymphocytes and promoting the anti-tumor activity of T lymphocytes.

### BRD7 regulated PD-L1 expression through the PI3K/AKT signaling pathway

**Figure 3:**
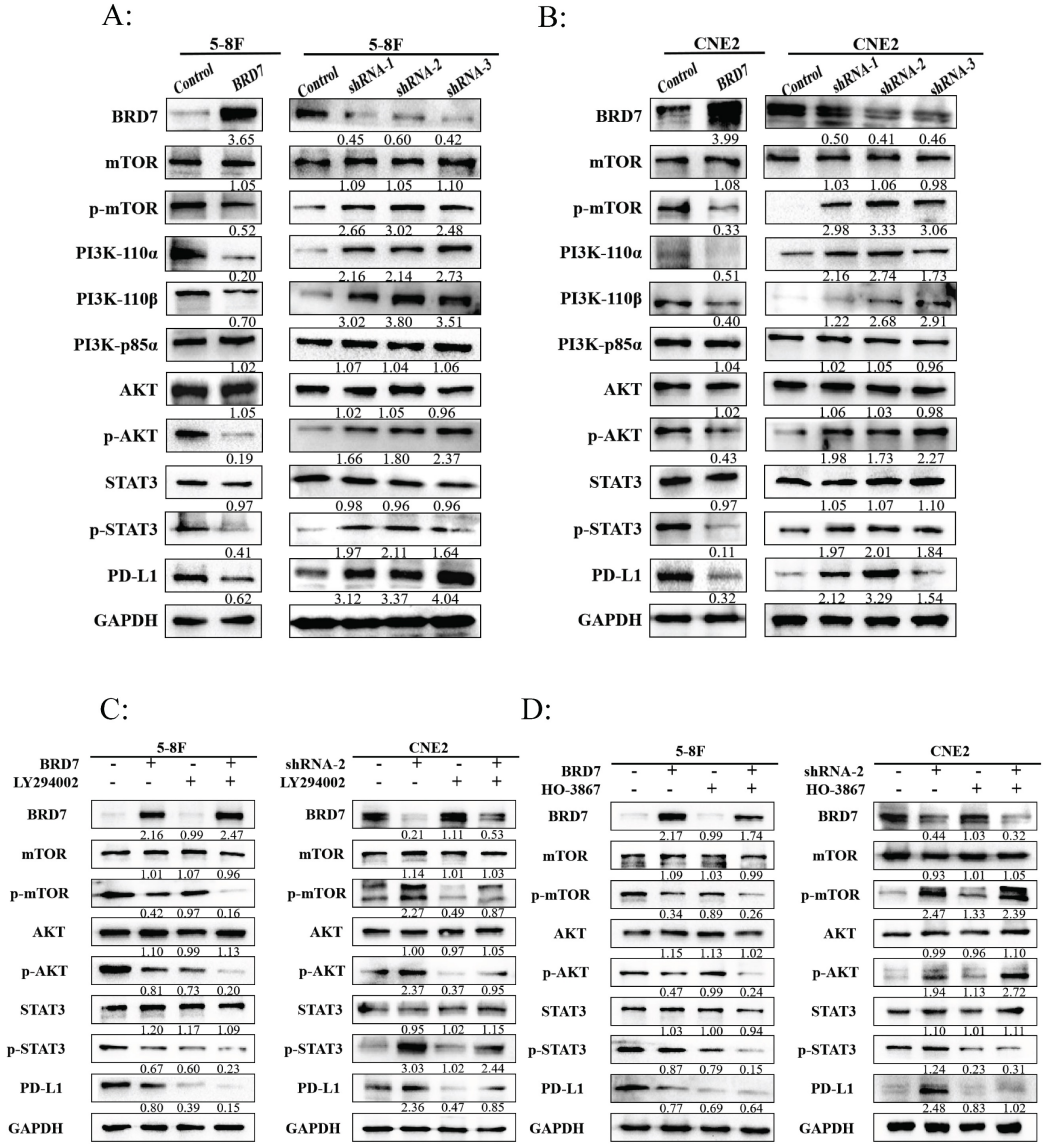
** BRD7 regulated PD-L1 expression through the PI3K/AKT signaling pathway. (**A-B) Representative western blot analysis of the expression of PI3K/AKT pathway molecules in NPC cell lines 5-8F (A) and CNE2 (B) stably transfected with BRD7 overexpression and knockdown or empty vector plasmids. (C-D) The PI3K inhibitor LY294002 (35μM) (C) or the p-STAT3 inhibitor HO-3867 (7μM). (D) was added to 5-8F cells with or without BRD7 overexpression and CNE2 cells with or without BRD7 knockdown. PD-L1 and the PI3K/AKT pathway molecules expression were detected by western blot.

To further investigate the regulatory relationship between BRD7 and PD-L1, we first demonstrated through IP-MS, Co-IP, and ChIP-qRCR (Figure S3A-C) that there was no direct interaction between BRD7 and PD-L1. Our previous studies had shown that the PI3K/AKT signaling pathway plays a crucial role in the invasion and metastasis of NPC 25. In addition, activated STAT3 regulates PD-L1 expression by binding to the PD-L1 promoter, and STAT3 could act as a downstream target of the PI3K/AKT signaling pathway. Does this pathway also participate in immune evasion in NPC? We transfected overexpression or interference plasmids of BRD7 and their controls into 5-8F and CNE2 cell lines. In the BRD7 overexpression group, the expression of p-AKT and p-mTOR proteins decreased, inhibiting the activation of the PI3K/AKT signaling pathway, while in the BRD7 interference group, this pathway was activated (Figure 3A-B). When BRD7 overexpression was combined with the PI3K inhibitor LY294002, the activation of the PI3K/AKT signaling pathway was further suppressed, leading to a further decrease in PD-L1 expression. However, LY294002 reversed the activation of the PI3K/AKT signaling pathway and the upregulation of PD-L1 expression caused by BRD7 interference (Figure 3C, Figure S1D). Subsequently, when BRD7 overexpression was combined with the p-STAT3 inhibitor HO-3867, the results showed that HO-3867 did not affect the activation of the PI3K/AKT signaling pathway but inhibited STAT3 phosphorylation and PD-L1 expression (Figure 3D, Figure S1E). Based on these results, we believe that BRD7 regulates PD-L1 expression in NPC cells by inhibiting the activation of the PI3K/AKT signaling pathway and suppressing STAT3 phosphorylation. We established a xenograft tumor model in nude mice using BRD7-interfered CNE2 cells and treated the tumor-bearing mice with LY-294002 and HO-3867, showing that downregulation of BRD7 expression promoted tumor growth *in vivo*, while treatment with LY-294002 and HO-3867 reversed the promoting effect of BRD7 downregulation on tumor growth (Figure S3D). Once again, we confirmed *in vivo* that BRD7 downregulated PD-L1 expression and inhibited NPC growth by inhibiting the activation of the PI3K/AKT/mTOR/STAT3 signaling pathway. (Figure S3E-F).

### BRD7 bound to PI3K-p85α and promoted the nuclear translocation of p85α

**Figure 4:**
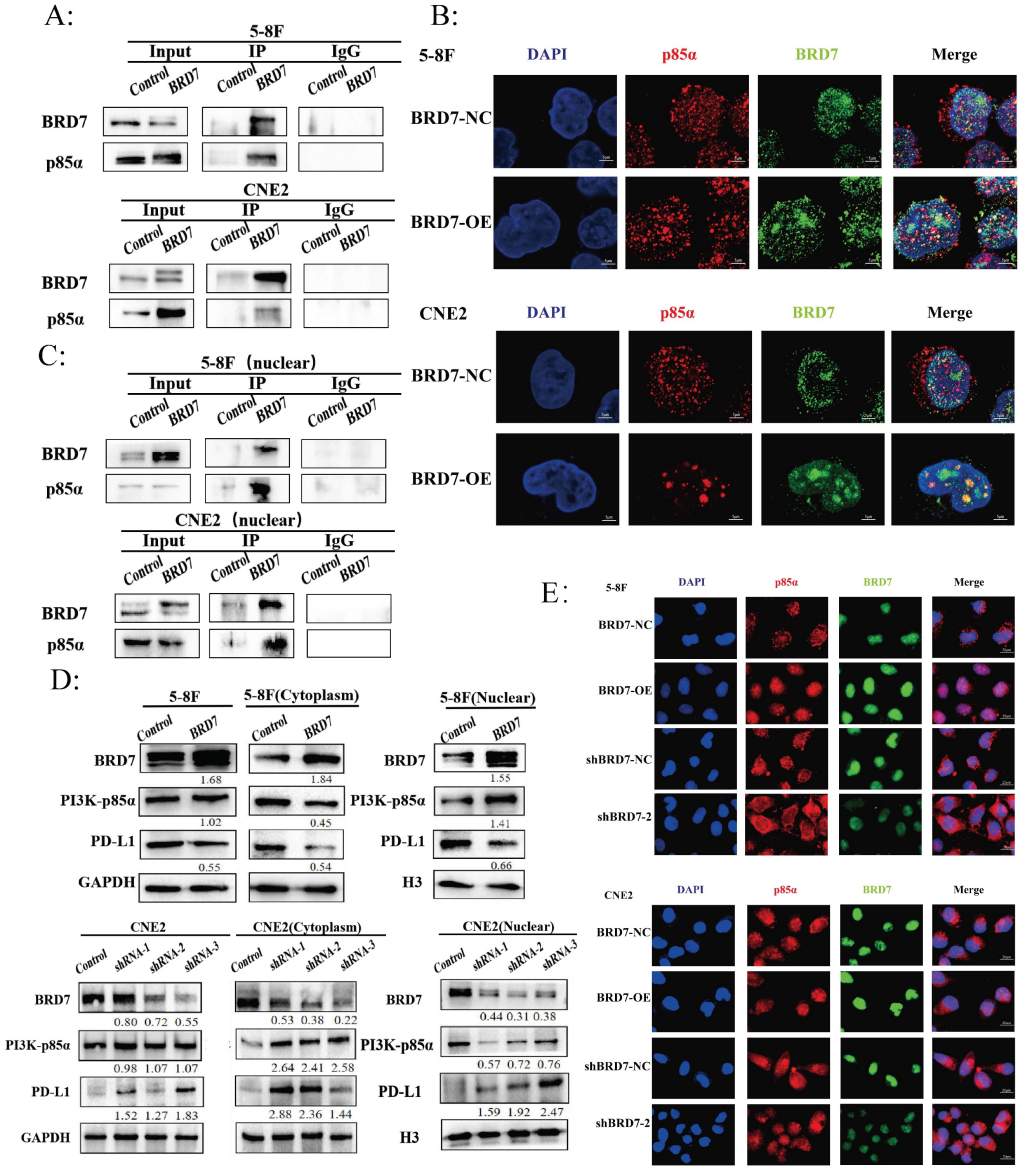
** BRD7 bound to PI3K-p85α and promoted the nuclear translocation of p85α.** (A) Co-IP assay detecting the association between BRD7 and p85α in total protein of 5-8F and CNE2 cells transfected with BRD7 pcDNA3.1-3xFlag-T2A-EGFP plasmid or the empty plasmids. (B) Immunofluorescence detecting the co-localization between BRD7 and p85α in 5-8F and CNE2 cells with or without BRD7 overexpression. The scale bar is 5 μm. (C) Co-IP assay detecting the association between BRD7 and p85α in the nucleus protein of 5-8F and CNE2 cells transfected with BRD7 pcDNA3.1-3xFlag-T2A-EGFP plasmid or the empty plasmids. (D) Representative western blot analysis of p85α and PD-L1 expression in total protein, the cytoplasm protein and the nucleus protein of 5-8F cells with or without BRD7 overexpression and CNE2 cells with or without BRD7 knockdown. (E) Immunofluorescence detecting the translocation of p85α in 5-8F and CNE2 cells with or without BRD7 overexpression or knockdown. The scale bar is 20 μm.

The Protein-Ligand Interaction Profiler (PLIP) was used to predict the interaction between BRD7 and p85α, with BRD7 as the reference chain, where purple represents BRD7 and yellow represents p85α. The HawkDock server calculated a binding free energy of -22.31 kcal/mol. There are 12 hydrogen bonds formed between the proteins (within 4.1Å) (Figure S4A-C). To further investigate the relationship between BRD7 and PI3K-p85α, we transfected the BRD7 plasmid into 5-8F and CNE2 cells and conducted Co-IP experiments (Figure 4A), which revealed their interaction. Observation under confocal laser microscopy showed that their binding primarily occurs in the cell nucleus (Figure 4B). Subsequent Co-IP experiments following nuclear-cytoplasmic protein separation confirmed that the interaction between BRD7 and PI3K-p85α mainly takes place in the cell nucleus (Figure 4C). PI3K-p85α is predominantly located in the cytoplasm, serving as a regulatory subunit that forms a heterodimer with the catalytic subunit p110, constituting PI3K. Why then is the localization of BRD7 with PI3K-p85α primarily in the cell nucleus? Western blot results indicated that when BRD7 is overexpressed, the total protein level of PI3K-p85α remains unchanged, decreases in the cytoplasm, and increases in the nucleus. Upon BRD7 interference, the total protein level of PI3K-p85α remained constant, increased in the cytoplasm, and decreased in the nucleus (Figure 4D). Furthermore, immunofluorescence experiments once again confirmed that when BRD7 was overexpressed, the fluorescence of PI3K-p85α in the nucleus is stronger. Conversely, when BRD7 was interfered with, the fluorescence of PI3K-p85α in the nucleus weakens (Figure 4E). These results suggested that BRD7 could interact with PI3K-p85α, promoting the nuclear translocation of p85α.

### BRD7 interacted with p85α in the 485-651 domain

**Figure 5:**
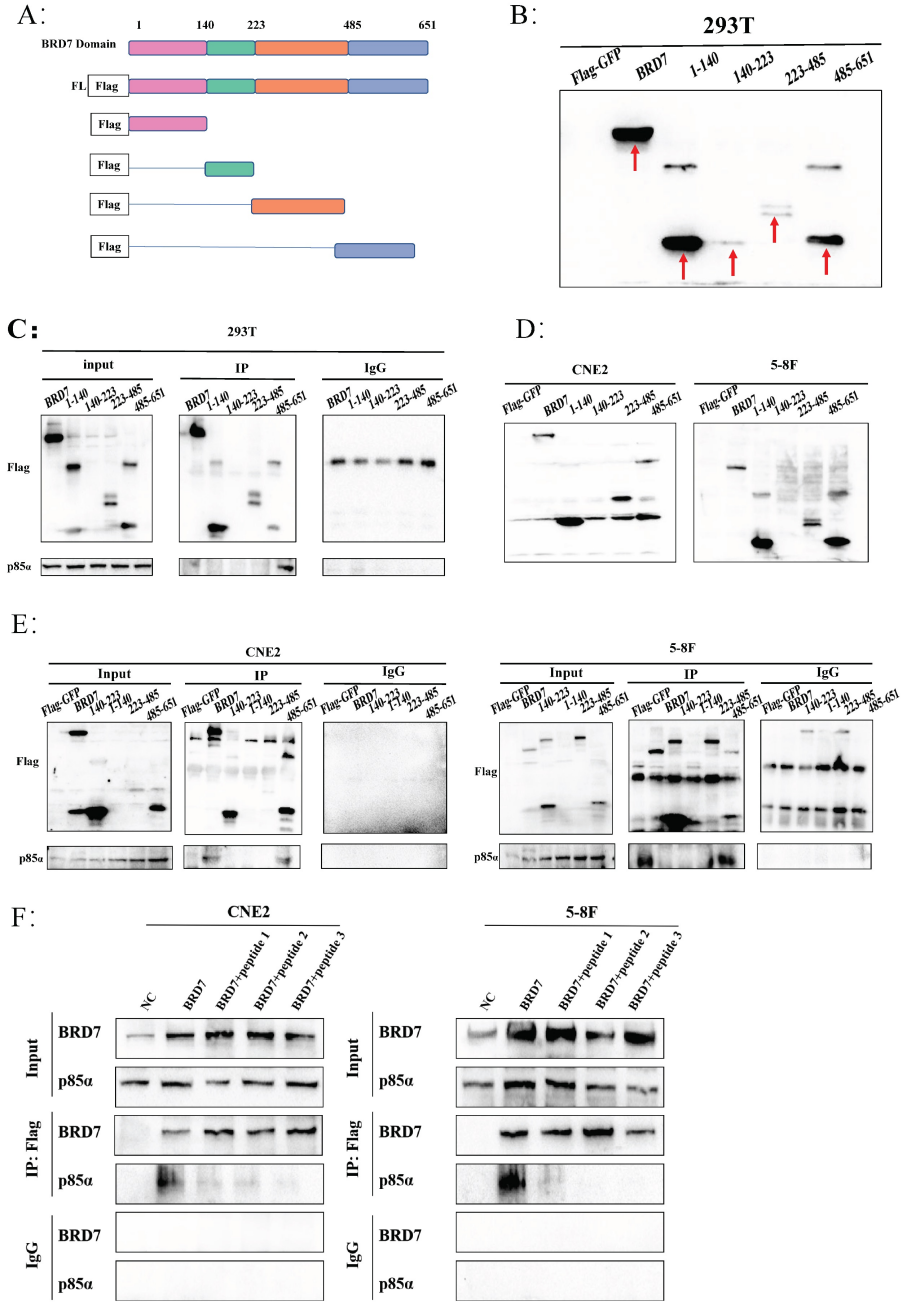
** BRD7 interacted with p85α in the 485-651 domain.** (A) Schematic representation of the construction of BRD7 truncation mutants. (B) Representative western blot analysis of verification of construction and stable transfection of BRD7 truncated mutants in 293T cells. (C) Co-IP assay detecting the association between BRD7 mutants and p85α in 293T cells stably transfected with BRD7 and its truncated mutants or empty vector plasmids. (D) Representative western blot analysis of verification of construction and stable transfection of BRD7 truncated mutants in 5-8F and CNE2 cells. (E) Co-IP assay detecting the association between BRD7 mutants and p85α in 5-8F and CNE2 cells stably transfected with BRD7 and its truncated mutants or empty vector plasmids. (F) In the 5-8F and CNE2 cell lines, Flag-GFP-BRD7 was overexpressed, and pull-down assays utilizing Flag revealed that the interaction between BRD7 and p85α was inhibited in the presence of peptides 1, 2, and 3 (5 μM).

To further explore the specific mechanism of BRD7 binding to PI3K-p85α, we initially analyzed the protein domain of BRD7 through the UniProt and PSIPRED Workbench (UCL.ac. UK) websites, constructed Flag-GFP-labeled plasmids containing different regions of BRD7 (BRD7-fl, 1-140, 140-223, 223-485, 485-651) (Figure 5A) and then validated them in 293T, CNE2, and 5-8F cells (Figure 5B, D). Combining specific domain studies of BRD7 and p85α, the Co-IP results indicated an interaction between BRD7 and p85α within the 485-651 domain (Figure 5C, E). Based on the molecular docking results and the 485-651 domain of BRD7, we designed three peptides to inhibit the interaction between BRD7 and p85α. The results demonstrated that peptides 1, 2, and 3 were all capable of blocking the binding of the two proteins. This finding provided further evidence that BRD7 exerts its function by interacting with p85α through the 485-651 domain (Figure 5F, Figure S4C).

### BRD7 could inhibit the immune escape of NPC

**Figure 6:**
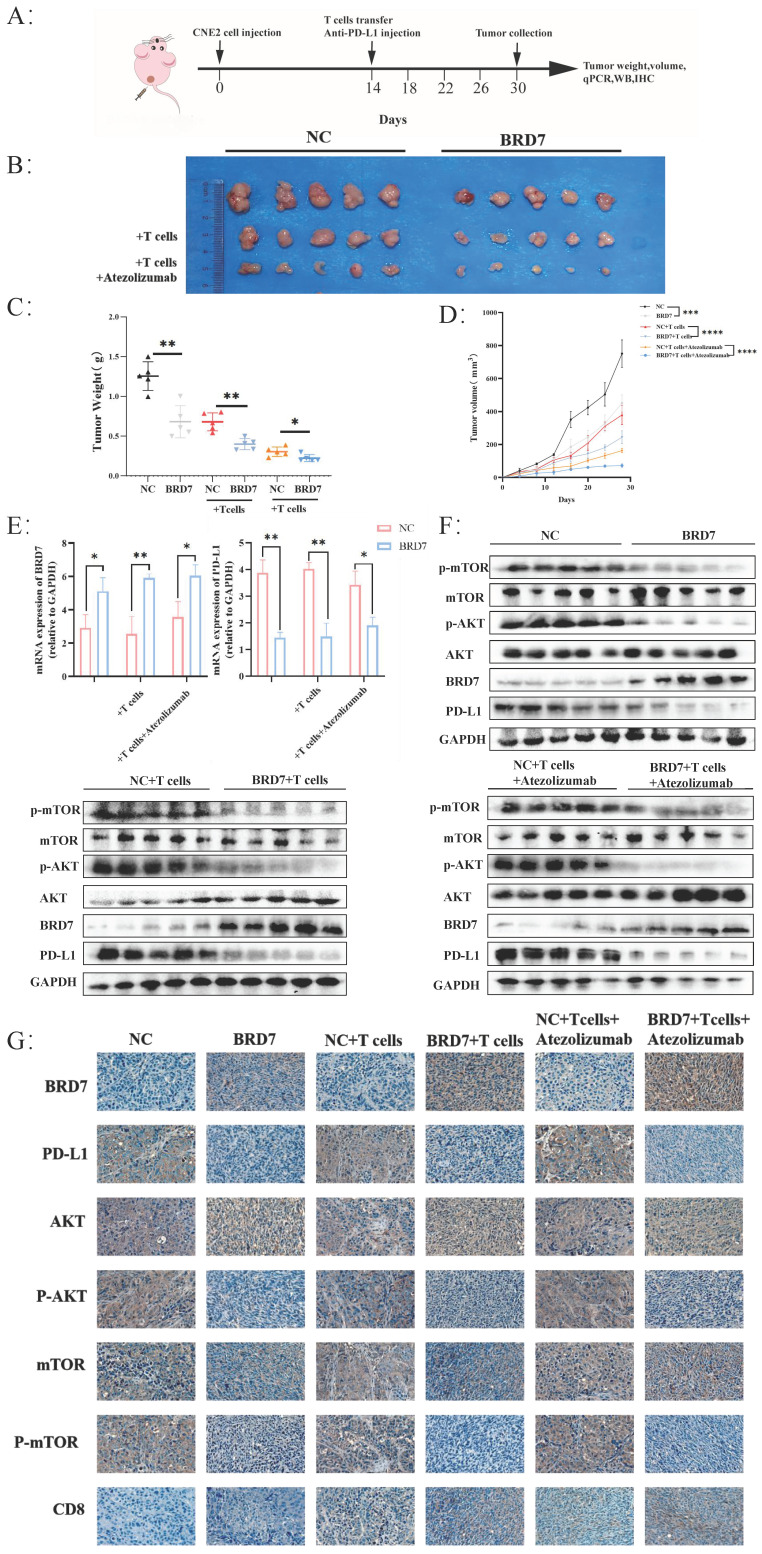
** BRD7 could inhibit the immune escape of NPC.** (A) Design of the experiment *in vivo*. (B) Images of the appearance of subcutaneous tumors in mice. n = 5 per group. (C) Statistical analysis of subcutaneous tumor weight. n = 5 per group. (D) Tumor growth curve. n = 5 per group. (E) Relative BRD7 and PD-L1 mRNA levels measured by q-PCR in tumor tissues. n = 5 per group. (F) Western blot analysis of BRD7, PD-L1, and PI3K/AKT pathway molecules in tumor tissues. (G) Representative images of immunohistochemical staining for BRD7, PD-L1, PI3K/AKT pathway molecules, and CD8 expression in tumor tissues. The scale bar is 20 μm. *, *P* < 0.05; **, *P* < 0.01; ***, *P* < 0.001; ****, *P* < 0.0001; ns, not significant.

To investigate whether BRD7 can inhibit NPC growth and immune escape *in vivo*, we established a xenograft tumor model in nude mice for *in vivo* experiments and applied adoptive T cell therapy to tumor-bearing mice (Figure 6A, Figure S5A). Overexpression of BRD7 can suppress tumor growth *in vivo*, and the inhibitory effect on tumor growth is more pronounced when BRD7 was overexpressed simultaneously with T cell injection, further enhanced by the use of Atezolizumab, which significantly inhibited tumor growth *in vivo* (Figure 6B-D). Tumor tissues from mice were collected to elucidate the molecular mechanisms by which BRD7 inhibited NPC growth and immune escape *in vivo*. Q-PCR results showed that the expression of PD-L1 in the BRD7 overexpression group was lower than that in the NC group (Figure 6E). Western blot analysis detected the expression of BRD7, PD-L1, and molecules related to the PI3K/AKT signaling pathway in tumor tissues (Figure 6F). The results indicated that the expression of p-AKT, p-mTOR, and PD-L1 in the BRD7 overexpression group was lower than in the corresponding NC group. IHC experiments using tumor tissue sections yielded consistent results (Figure 6G, Figure S5B). To assess the impact of BRD7 on anti-tumor immunity in NPC, we examined the infiltration of CD8^+^ T lymphocytes in tumor tissues (Figure 6G, Figure S5B). As shown, BRD7 promoted the infiltration of CD8^+^ T lymphocytes, thereby enhancing their cytotoxicity against tumor cells. Furthermore, the addition of Atezolizumab treatment led to further increased infiltration of CD8^+^ T lymphocytes. A co-transfection model of BRD7 and PD-L1 was established in CNE2 cells, followed by *in vivo* experiments in nude mice. The results demonstrated that PD-L1 partially reversed the anti-tumor effect of BRD7 *in vivo* (Figure S5C-E), reaffirming that BRD7 could promote T cell-mediated killing of tumor cells by downregulating PD-L1 expression, thereby inhibiting immune escape in NPC.

## Discussion

The treatment of NPC with immune checkpoint inhibitors (ICIs) is currently a hot research topic. Effective ICIs can break through the barrier of immune defense, restore endogenous anti-tumor immunity, and the most representative ICI targets are PD-1/PD-L1 and CTLA-4 26. Further exploration of immune escape and PD-L1 regulatory mechanisms in NPC can provide new insights to enhance the efficacy of clinical immunotherapy. In this study, our results demonstrated that BRD7 could inhibit immune escape through down-regulating PD-L1 expression in NPC for the first time.

BRD7 is a member of the bromodomain protein family and was first identified as a candidate tumor suppressor gene for NPC in 2001 27. Since the initial discovery of decreased BRD7 expression levels in NPC, its role has attracted researchers' attention. Studies had shown that BRD7 acted as a tumor suppressor gene at different stages of NPC. Overexpression of BRD7 could inhibit NPC cell proliferation, block the G1 to S phase cell cycle progression through the Ras/MEK/ERK, PI3K/AKT, and Rb/E2F signaling pathways, induce cell apoptosis, and reverse the malignant phenotype of NPC cells 28-31. Additionally, BRD7 was downregulated in various cancers, such as breast cancer, cervical cancer, ovarian cancer, and gastric cancer 32-35. However, it is worth noting that Zhao *et al.* found that BRD7 plays a pro-inflammatory role in advanced colorectal cancer and may have carcinogenic effects, which is different from its inhibitory effects in other tumors, suggesting that BRD7 may have a dual role as a tumor suppressor and oncogene 36, 37. These pieces of evidence indicated that BRD7 played a significant role in various physiological and pathological conditions. However, the relationship between BRD7 and immune escape has not been reported.

PD-L1 plays a crucial role in immune escape of tumor cells, which is often expressed on the surface of tumor cells and immune inhibitory cells in the tumor microenvironment (TME) and interacts with PD-1 expressed on T cells. The PD-1/PD-L1 immune checkpoint can prevent T cells from being effectively activated and killing tumor cells, mediating tumor immune escape. Therefore, anti-PD-1/PD-L1 or blockade of the PD-1/PD-L1 axis can significantly improve the treatment outcomes of advanced cancer patients by promoting the reactivation of immune cells 38. ICIs based on PD-1/PD-L1 blockade (such as pembrolizumab and camrelizumab) were approved in China in 2021 for the treatment of refractory, recurrent and/or metastatic NPC 39. Although several PD-1/PD-L1 antagonists have been approved by the FDA and are in clinical trials, their therapeutic effects on solid tumors have not met expectations. In PD-L1-positive metastatic melanoma and lung cancer, the efficacy of anti-PD-L1 antagonists is only 40%-50%; in colorectal cancer, although the PD-L1 positivity rate is as high as 50%, the efficacy of anti-PD-1 or anti-PD-L1 therapy is still not optimistic 38, 40-42; this is closely related to the complex tumor microenvironment. Considering the key role of PD-1 in regulating immune escape of tumor cells, we also used an *in vitro* co-culture system of NPC cells and T cells. We found that overexpression of BRD7 in tumor cells inhibited the apoptosis of CD8^+^ T lymphocytes in the co-culture system, reduced the proportion of PD-1^+^ T cells, and increased IFN-γ secretion. This indicates an enhanced cytotoxic effect of T cells on NPC cells and a suppression of immune escape mechanisms. And BRD7 combined with PD-L1 antibody showed a better anti-tumor effect.

To investigate the mechanism by which BRD7 regulates PD-L1 expression, we performed ChIP, IP-MS, and Co-IP experiments, which confirmed that BRD7 could not directly downregulate PD-L1 expression. In our preliminary research, we found that activation of the PI3K/AKT signaling pathway is involved in the occurrence of EMT in NPC 25. PI3K/AKT/mTOR is an important intracellular signaling pathway involved in regulating the occurrence and development of cancer, including cell metabolism, cell proliferation, cell apoptosis, and immune monitoring of the tumor microenvironment 43-45. Transcription factors, including c-MYC and STAT3, can regulate the expression of PD-L1 at the transcriptional level 46-49. In addition, STAT3 has been confirmed as a downstream target of the AKT/mTOR signaling pathway 50, 51. Therefore, we reasonably speculate whether BRD7 inhibits STAT3 activation by downregulating the activity of the PI3K/AKT/mTOR signaling pathway, thereby suppressing PD-L1 expression at the transcriptional level. We conducted experiments on NPC cells and treated them with the PI3K inhibitor LY294002 and the p-STAT3 inhibitor HO-3867. The results showed that BRD7 could indeed downregulate PD-L1 expression by inhibiting the activity of this pathway. In addition, we predicted that BRD7 may interact with PI3K-p85α. Further Co-IP and immunofluorescence assays confirmed that BRD7 could bind to PI3K-P85α and induce its nuclear translocation through the 485-651 domain of BRD7.

Although our study suggested that BRD7 could serve as an effective target to inhibit the growth of NPC and promote anti-tumor immunity *in vivo*, this study still has certain limitations. Further research is needed to investigate the mechanism of BRD7 affects T cells when it is overexpression in NPC cells. If BRD7 could downregulate the PD-1 levels of T cells, the mechanism of T cells internally regulates their killing function against tumor cells still needs further exploration. Currently, due to the lack of mature *in situ* mouse models and mouse-derived NPC cell lines, the mechanisms of NPC immune escape can only be achieved through establishing a transplant tumor model in immunodeficient mice, which cannot simulate a normal immune environment, posing an urgent issue to be addressed. The tumor microenvironment not only includes tumor cells and T lymphocytes but also innate immune cells, cytokines, and antigen-presenting cells (APCs) such as NK cells, dendritic cells, and macrophages 52. These APCs also inhibit T cell activation by expressing PD-L1 and interacting with PD-1 on T cells. At the same time, they can secrete various cytokines such as IFN-γ, IL-2, IL-10, indirectly promoting the expression of PD-L1 in tumor cells and APCs 53, 54. In this study, we found that BRD7 could inhibit the expression of PD-L1 in tumor cells by downregulating the activity of the PI3K/AKT signaling pathway, thereby inhibiting its binding to PD-1 on T cells, restoring the cytotoxic function of CD8^+^ T cells. However, whether BRD7 affects other immune checkpoints and the expression of PD-L1 on APCs requires further research. The role of BRD7 in NPC immune escape still needs further exploration.

## Conclusions

**Figure 7:**
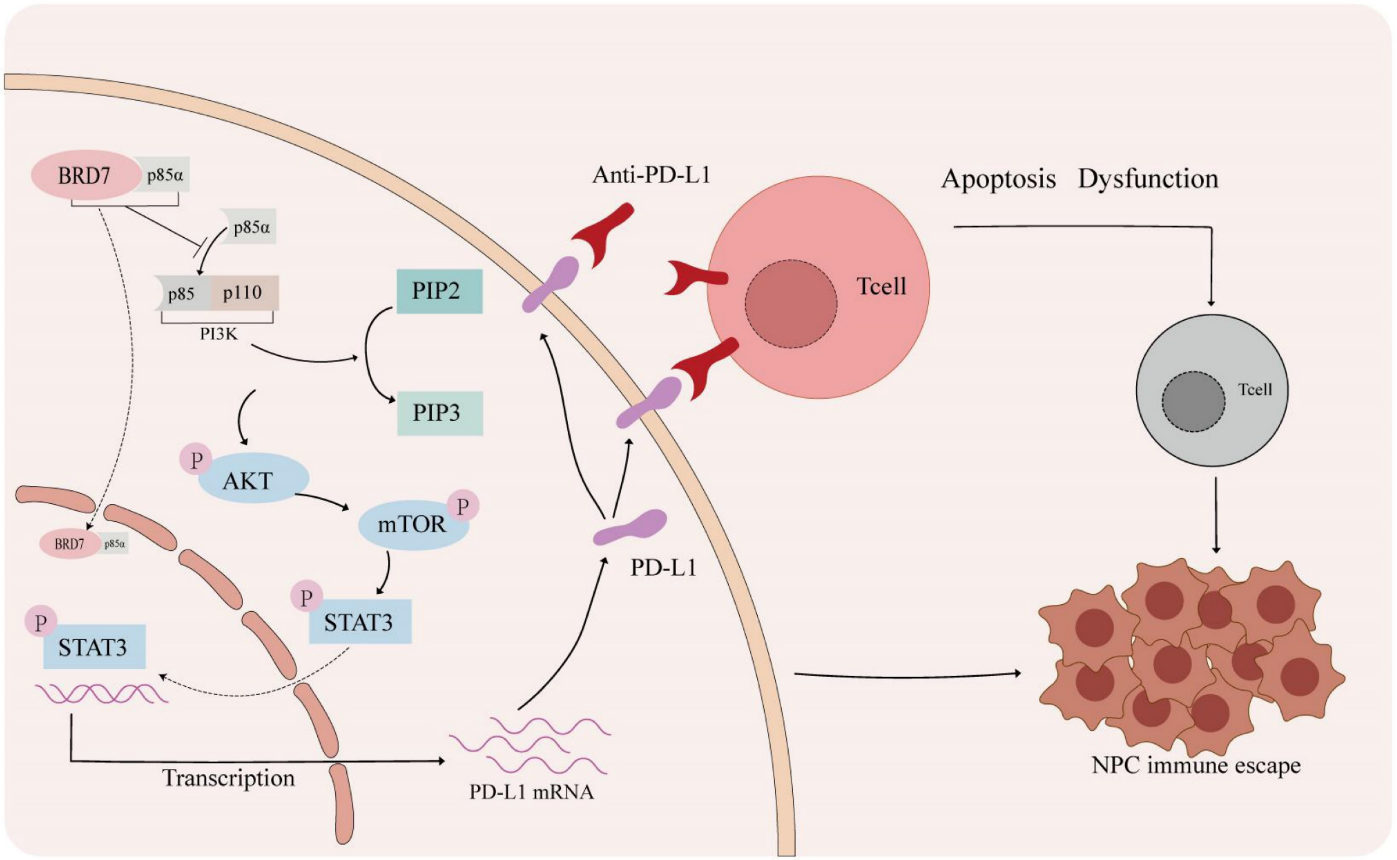
** The mechanism of BRD7 inhibiting immune escape of NPC.** In the tumor microenvironment of nasopharyngeal carcinoma, activation of the PD-1/PD-L1 axis can lead to T cell apoptosis and dysfunction. Blocking the interaction between PD-1 and PD-L1 with a PD-L1 antibody can help prevent this. BRD7 can enhance the nuclear translocation of p85α by binding to it, inhibiting the PI3K/AKT/mTOR/STAT3 signaling pathway and reducing PD-L1 expression to prevent immune evasion in nasopharyngeal carcinoma.

BRD7 could inhibit NPC growth and enhance the cytotoxicity of CD8^+^ T lymphocytes through downregulating PD-L1 expression, thereby suppressing immune escape in NPC (Figure 7).

## Supplementary Material

Supplementary figures and tables.

## Abbreviations

NPC: nasopharyngeal carcinoma
CSCO: Chinese Society of Clinical Oncology
PD-1: programmed cell death-1
PD-L1: programmed cell death-Ligand 1
BRD7: bromodomain-containing 7
GEO: Gene Expression Omnibus
NPE: nasopharyngeal epithelium
ChIP: Chromatin immunoprecipitation
q-PCR: quantitative PCR
WB: Western blot
Co-IP: Co-immunoprecipitation
IHC: immunohistochemistry
DFS: disease-free survival
OS: overall survival
PLIP: Protein-Ligand Interaction Profiler
ICIs: immune checkpoint inhibitors
TME: tumor microenvironment
APCs: antigen-presenting cells
