# Supplementary material for: BRD7 Inhibited Immune Escape in Nasopharyngeal Carcinoma via Inhibiting PD-L1 Expression
Source: Int J Biol Sci. 2025 Feb 10;21(5):1914–31. doi: 10.7150/ijbs.103703 (PMC11900823; doi:10.7150/ijbs.103703)
Supplement: Supplementary file 1 — Supplementary figures and tables. [file ijbsv21p1914s1.pdf]

S1A:

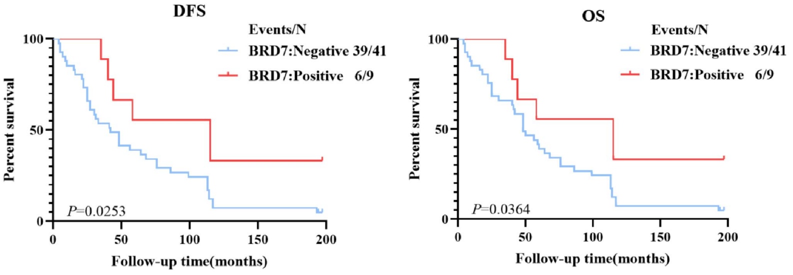

S1B:

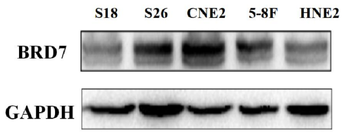

S1C:

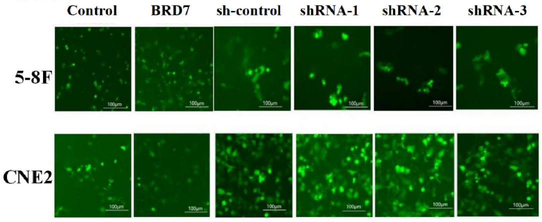

S1D:

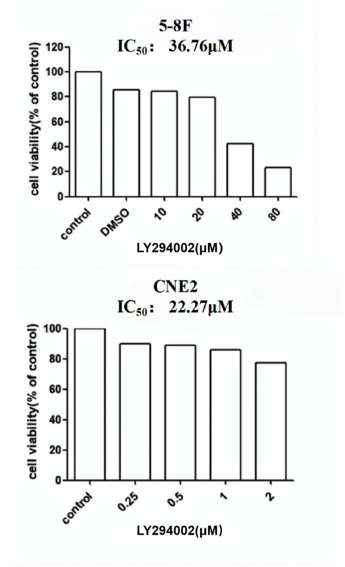

S1E:

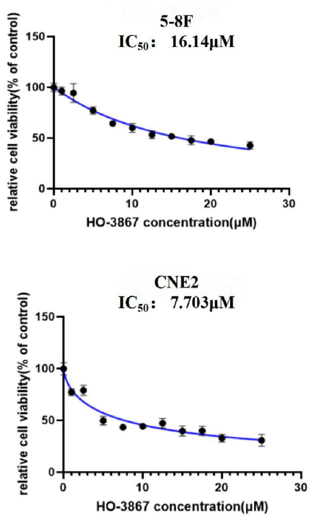

Figure S1 Survival curve of 50 patients with NPC. Verification of overexpression and knockdown effects of BRD7. IC50 value of PI3K and p-STAT3 inhibitors.

A. Kaplan-Meier survival curve of 50 follow-up patients. BRD7 was associated with OS and DFS. OS: BRD7 positive (red), BRD7 negative (blue), *P*=0.0364, log-rank test. DFS: BRD7 positive (red), BRD7 negative (blue), *P*=0.0253, log-rank test.

B. Background expression of NPC cell lines S18, S26, HNE2, CNE2, and 5-8F.

C. Representative images of immunofluorescence indicating plasmids transfection effect. The scale bar is 100 μm.

D. IC50 value of PI3K inhibitor LY294002 in 5-8F and CNE2 cells.

E. IC50 value of p-STAT3 inhibitor HO-3867 in 5-8F and CNE2 cells.

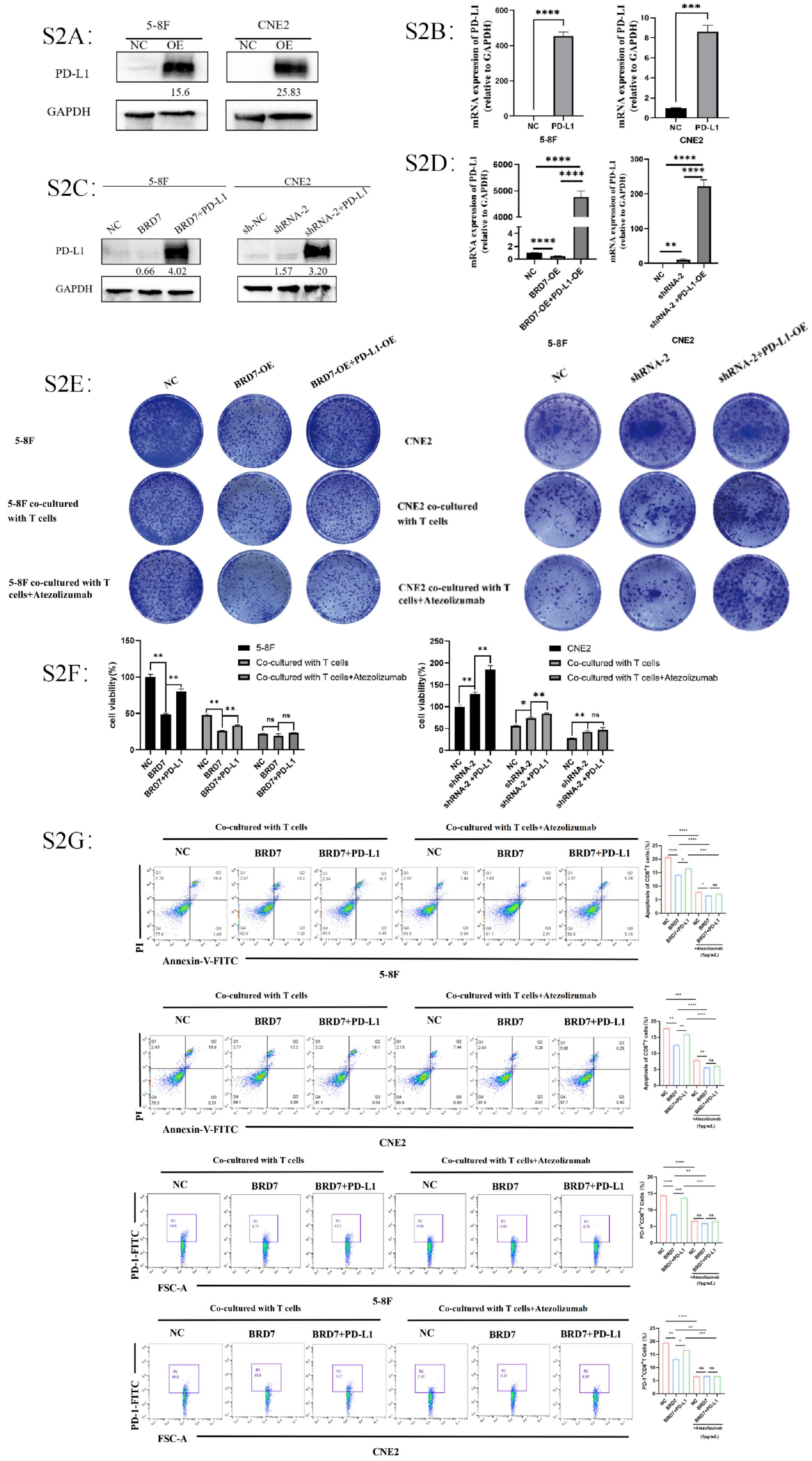
S2A:
5-8F
CNE2
NC
OE
PD-L1
GAPDH
15.6
25.83
S2B:
mRNA expression of PD-L1 (relative to GAPDH)
****
***
NC
PD-L1
5-8F
CNE2
S2C:
BRD7
BRD7+PD-L1
sh-NC
shRNA-2
shRNA-2+PD-L1
0.66
4.02
1.57
3.20
S2D:
BRD7-OE
BRD7-OE+PD-L1-OE
shRNA-2+PD-L1-OE
S2E:
shRNA-2+PD-L1-OE
5-8F co-cultured with T cells
5-8F co-cultured with T cells+Atezolizumab
CNE2 co-cultured with T cells
CNE2 co-cultured with T cells+Atezolizumab
S2F:
cell viability(%)
Co-cultured with T cells
Co-cultured with T cells+Atezolizumab
ns
S2G:
Co-cultured with T cells+Atezolizumab
PI
Annexin-V-FITC
Apoptosis of CD8+T cells(%)
PD-1-FITC
FSC-A
PD-1+CD8+T Cells (%)
+Atezolizumab
(5μg/mL)

Figure S2 BRD7 downregulated PD-L1 expression and enhanced the cytotoxicity of T lymphocytes against tumor cells.

A. PD-L1 expression measured by western blot in 5-8F and CNE2 cells stably transfected with PD-L1 overexpression or empty vector plasmids.
B. Relative PD-L1 mRNA levels measured by q-PCR in 5-8F and CNE2 cells stably transfected with PD-L1 overexpression or empty vector plasmids.
C. PD-L1 expression measured by western blot in 5-8F cells transfected with BRD7 and PD-L1 overexpression plasmids and in CNE2 cells transfected with BRD7 knockdown and PD-L1 overexpression plasmids.
D. Relative PD-L1 mRNA levels measured by q-PCR in 5-8F cells transfected with BRD7 and PD-L1 overexpression plasmids and in CNE2 cells transfected with BRD7 knockdown and PD-L1 overexpression plasmids.
E. Clonogenic assays of 5-8F cells transfected with BRD7 and PD-L1 overexpression plasmids and CNE2 cells transfected with BRD7 knockdown and PD-L1 overexpression plasmids with or without T cells co-culture and PD-L1 antibody incubation. Atezolizumab: the PD-L1 antibody.
F. CCK-8 assay of 5-8F cells transfected with BRD7 and PD-L1 overexpression plasmids and CNE2 cells transfected with BRD7 knockdown and PD-L1 overexpression plasmids with or without T cells co-culture and PD-L1 antibody incubation. Absorbance values were detected at 450 nm.
G. Flow cytometry detecting the apoptosis ratio of $CD8^+$ T cells and the ratio of $PD\text{-}1^+$ $CD8^+$ T cells in T cells co-cultured with 5-8F and CNE2 cells transfected with BRD7 and PD-L1 overexpression plasmids with or without PD-L1 antibody incubation.*, $P < 0.05$; **, $P < 0.01$; ***, $P < 0.001$; ****, $P < 0.0001$; ns, not significant.

S3A:

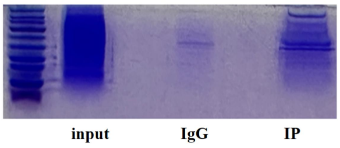

S3B:

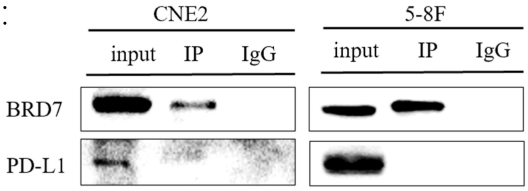

S3C:

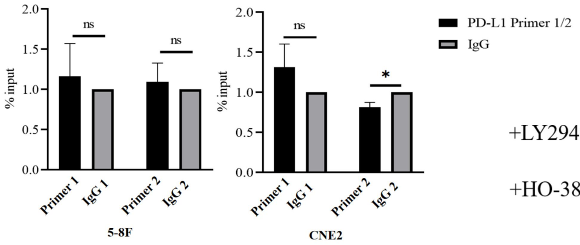

S3D:

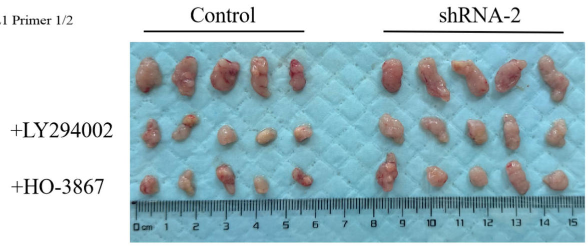

S3E:

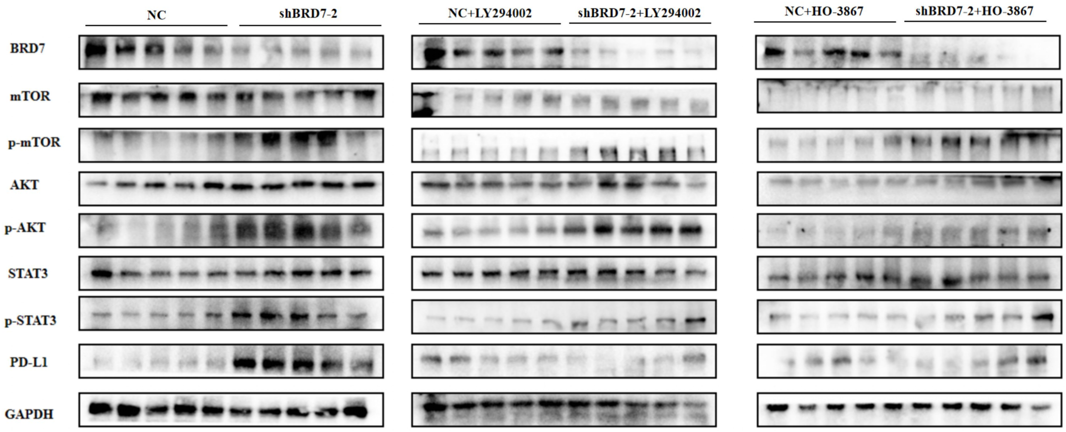

S3F:

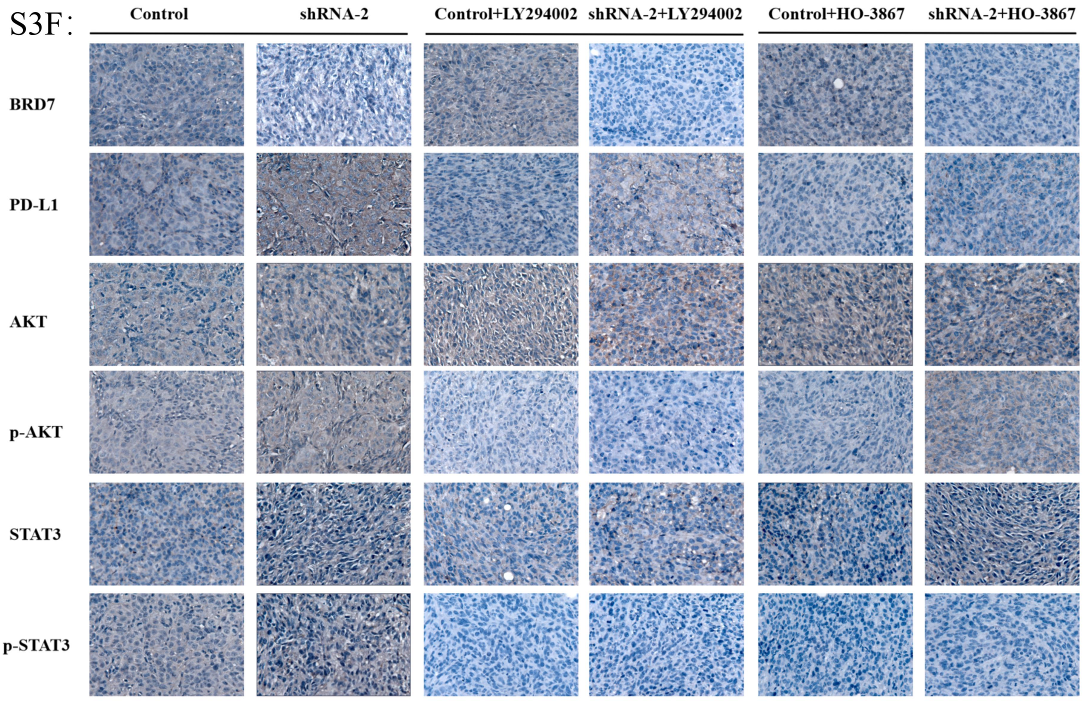

Figure S3 BRD7 inhibited the PI3K/AKT/mTOR/STAT3 signaling pathway to downregulate PD-L1 expression, thereby suppressing nasopharyngeal carcinoma growth.

A. BRD7 is highly expressed in CNE2 cells. CNE2 cells were harvested and subjected to immunoprecipitation with BRD7 antibody, followed by gel strip and mass spectrometry analysis.
B. The interaction between BRD7 and PD-L1 at the protein level was detected by Co-IP assay. In the two cell lines, the BRD7 antibody could pull down the BRD7 protein but could not pull-down PD-L1.
C. The BRD7 pcDNA3.1-3xFlag-T2A-EGFP plasmid was transfected into 5-8F and CNE2 cell lines. ChIP-qPCR was performed with FLAG antibody and mouse IgG antibody. Compared with the IgG group, BRD7 couldn't directly bind to the PD-L1 promoter.
D. Images of the appearance of subcutaneous tumors in mice. n = 5 per group.
E. Western blot analysis of BRD7, PD-L1, and PI3K/AKT/STAT3 pathway molecules in tumor tissues.
F. Representative images of immunohistochemical staining for BRD7, PD-L1, and PI3K/AKT/STAT3 pathway molecules. The scale bar is 20 μm. *, $P < 0.05$; **, $P < 0.01$; ***, $P < 0.001$; ****, $P < 0.0001$; ns, not significant.

S4A:

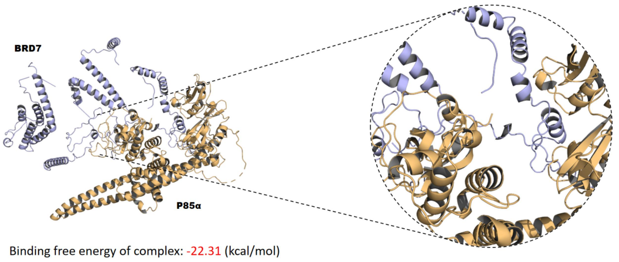

Binding free energy of complex: -22.31 (kcal/mol)

Summary of the Top 10 Models

Rank	1	2	3	4	5	6	7	8	9	10
Docking Score	-248.66	-246.06	-236.61	-229.85	-225.71	-224.01	-221.76	-219.59	-217.21	-215.54
Confidence Score	0.8780	0.8723	0.8497	0.8316	0.8197	0.8146	0.8077	0.8009	0.7932	0.7876

S4B:

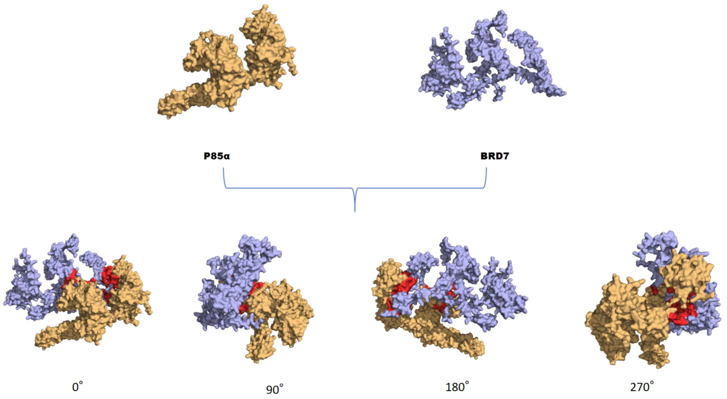

S4C:

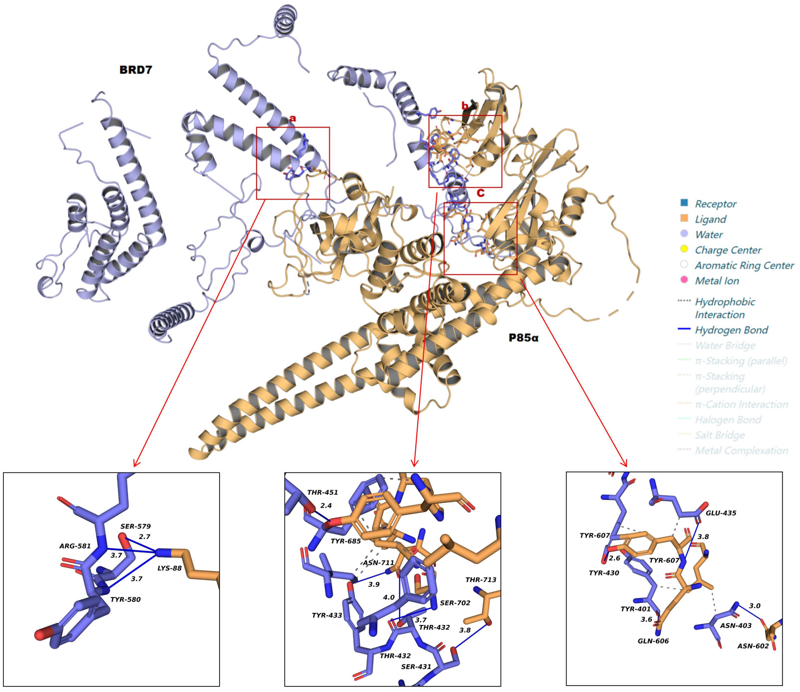

a b c

Figure S4 Molecular docking of BRD7 and p85α.

A. Using PLIP to predict the interaction between BRD7 and p85α, with BRD7 as the reference chain. Purple represents BRD7 and yellow represents p85α. The HawkDock server calculated the binding free energy to be -22.31 kcal/mol, with 12 hydrogen bonds formed between the proteins within 4.1Å.

B-C: Schematic diagram of BRD7 binding to p85α.

S5A:

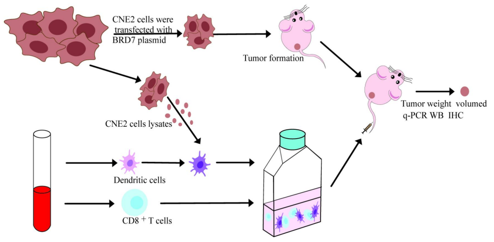

S5C:

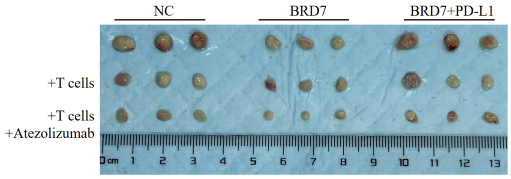

S5B:

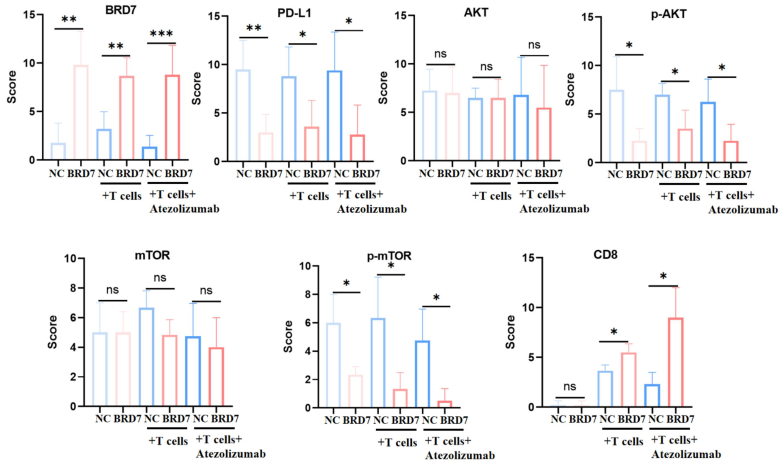

S5D:

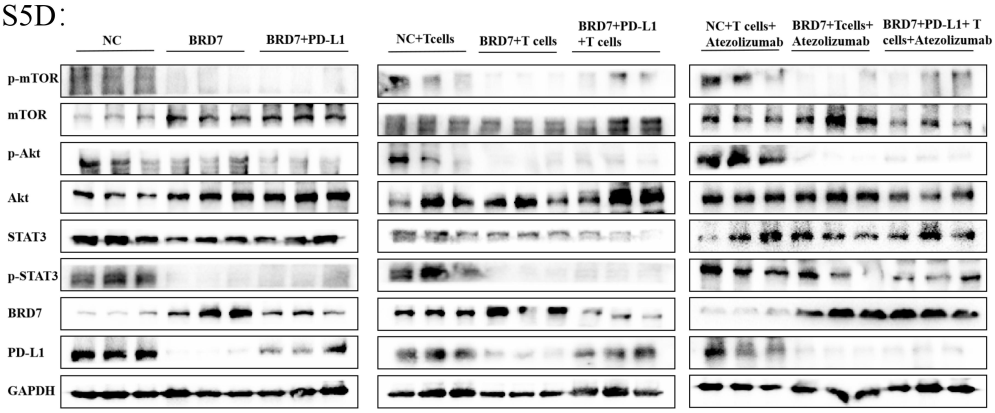

S5E:

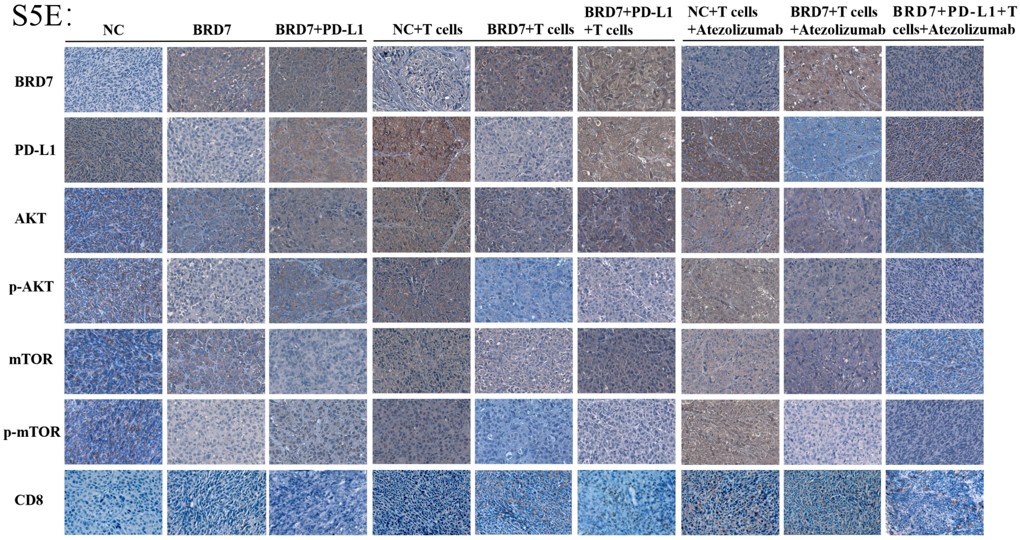

Figure S5 PD-L1 partially reversed the anti-tumor effect of BRD7 in vivo.

A. Flow chart of animal experiment.

B. The score of immunohistochemical staining for BRD7, PD-L1, PI3K/AKT pathway molecules, and CD8 expression in tumor tissues.

C. Images of the appearance of subcutaneous tumors in mice. n = 3 per group.

D. Western blot analysis of BRD7, PD-L1, and PI3K/AKT/STAT3 pathway molecules in tumor tissues.

E. Representative images of immunohistochemical staining for BRD7, PD-L1, PI3K/AKT pathway molecules, and CD8 expression in tumor tissues. The scale bar is 20 μm.*, $P < 0.05$; **, $P < 0.01$; ***, $P < 0.001$; ****, $P < 0.0001$; ns, not significant.

Table.S1 mRNA primer sequences

	**Forward**	**Reverse**
BRD7	AGCCAGGCTACTGCCCTG	GGAGTCCAAACGCCCTGGT
PD-L1	CAATTTGTGCATGGAGAGGAAG	GTTGTATGGGGCATTGACTTTC
GAPDH	GCATTGCCCTCAACGACCAC	CCACCACCCTGTTGCTGTAG
Primer1	TGCGTTCAGATGTTGGCTTGTTG	CCGGGAAGAGTTTCGAAGATTAA AGC
Primer2	CACCTACTTTCTAGAATAAAAAC CAAAGCC	GCCTCTTCAAGGTGACTGAACAT C

Table.S2 Major antibody

Antibody	source
Rabbit anti-Human BRD7 Antibody	Proteintech
Rabbit anti-Human PD-L1 Antibody	Proteintech
Rabbit anti-Human p-mTOR Antibody	AiFang Biological /Abmart
Rabbit anti-Human mTOR Antibody	CST/servicebio
Rabbit anti-Human PI3K-p85 Antibody	Proteintech
Rabbit anti-Human PI3K-p85α Antibody	Proteintech
Mouse anti-Human PI3K-p110α Antibody	Abmart
Mouse anti-Human PI3K-p110β Antibody	ZEN-BIOSCIENCE
Rabbit anti-Human p-AKT Antibody	Proteintech/Abmart
Rabbit anti-Human AKT Antibody	Proteintech/ZEN-BIOSCIE NCE
Rabbit anti-Human STAT3 Antibody	Proteintech
Rabbit anti-Human p-STAT3 Antibody	ZEN-BIOSCIENCE
Rabbit anti-Human Histone H3 Antibody	Abcam
Goat anti-Rabbit IgG(H+L) Highly Cross-Adsorbed Secondary Antibody	Proteintech
Goat anti-Mouse IgG(H+L) Highly Cross-Adsorbed Secondary Antibody	Proteintech
Rabbit anti-Human GAPDH Antibody	Proteintech
APC Anti-Human CD3 Antibody	Elabscience
PerCP Anti-Human CD8a Antibody	Elabscience
Rabbit/Mouse IgG	Beyotime Biotechnology
FITC Anti-Human CD279/PD-1 Antibody	Elabscience
FITC Anti-Human PD-1 Antibody	Elabscience

Table.S2 (continued) Major antibody

Antibody	source
Annexin V-FITC/PI Apoptosis Assay kit	Biosharp
Mouse anti-Human CD8 Antibody	Santa
DYKDDDDK tag Polyclonal antibody (Binds to FLAG® tag epitope)	Proteintech
DYKDDDDK tag Monoclonal antibody (Binds to FLAG® tag epitope)	Proteintech
Goat Anti-Mouse IgG H&L (Alexa Fluor® 594)	Abcam
Goat Anti-Rabbit IgG H&L (Alexa Fluor® 488)	Abcam
Atezolizumab	GlpBio

Table.S3 Relationship between BRD7 and clinicopathological features

**Clinicopathological characteristics**	**BRD7 +/-**	**P value**
Age：		
≤48	21/153	0.011
>48	39/135	
Gender:		
male	54/212	0.007
female	6/76	
T:		
T1+T2	36/174	0.995
T3+T4	24/111	
M:		
metastasis	44/186	0.203
No metastasis	16/101	
Clinical stage:		
Stages I+II	22/118	0.497
Stages III+IV	38/167	
Recurrence:		
No recurrence	12/6	0.001
recurrence	4/21	
WHO classification：		
WHO Ia	3/12	0.736 (P^{a-b} value)
WHO IIb	57/265	0.221(P^{b-c} value)
WHO IIIc	0/10	

Table.S4 Polypeptide sequence

	**sequences**(N->C)	**purity**
peptide 1	RLSTRPPPNMICLLGPSYREM	95%
peptide 2	LGPSYREMHLAEQVTNNLKELA	95%
peptide 3	RLSTRPPPNMICLLGPSYRE	95%
